# Fluctuating methylation clocks for cell lineage tracing at high temporal resolution in human tissues

**DOI:** 10.1038/s41587-021-01109-w

**Published:** 2022-01-03

**Authors:** Calum Gabbutt, Ryan O. Schenck, Daniel J. Weisenberger, Christopher Kimberley, Alison Berner, Jacob Househam, Eszter Lakatos, Mark Robertson-Tessi, Isabel Martin, Roshani Patel, Susan K. Clark, Andrew Latchford, Chris P. Barnes, Simon J. Leedham, Alexander R. A. Anderson, Trevor A. Graham, Darryl Shibata

**Affiliations:** 1 Evolution and Cancer Laboratory, Centre for Genomics and Computational Biology, Barts Cancer Institute, Barts and the London School of Medicine and Dentistry, Queen Mary University of London, London, UK; 2 Department of Cell and Developmental Biology, University College London, London, UK; 3 London Interdisciplinary Doctoral Training Programme (LIDo), London, UK; 4 Integrated Mathematical Oncology Department, Moffitt Cancer Center, Tampa, FL USA; 5 Intestinal Stem Cell Biology Lab, Wellcome Centre for Human Genetics, University of Oxford, Oxford, UK; 6 Department of Biochemistry and Molecular Medicine, University of Southern California, Los Angeles, CA USA; 7 St. Mark’s Hospital, Harrow, London, UK; 8 Department of Surgery and Cancer, Imperial College, London, UK; 9 Department of Pathology, Keck School of Medicine, University of Southern California, Los Angeles, CA USA

**Keywords:** Computational models, Intestinal stem cells, Bioinformatics, Intestinal stem cells

## Abstract

Molecular clocks that record cell ancestry mutate too slowly to measure the short-timescale dynamics of cell renewal in adult tissues. Here, we show that fluctuating DNA methylation marks can be used as clocks in cells where ongoing methylation and demethylation cause repeated ‘flip–flops’ between methylated and unmethylated states. We identify endogenous fluctuating CpG (fCpG) sites using standard methylation arrays and develop a mathematical model to quantitatively measure human adult stem cell dynamics from these data. Small intestinal crypts were inferred to contain slightly more stem cells than the colon, with slower stem cell replacement in the small intestine. Germline *APC* mutation increased the number of replacements per crypt. In blood, we measured rapid expansion of acute leukemia and slower growth of chronic disease. Thus, the patterns of human somatic cell birth and death are measurable with fluctuating methylation clocks (FMCs).

## Abstract (web summary)

Lineage tracing of human stem cells is achieved by measuring fluctuating DNA methylation.

## Main

The fates of individual human cells in vivo are difficult to reconstruct. In animal models, the use of transgenic or exogenous cell labeling enables straightforward clonal lineage tracing^1–10^, but in humans, these methods are precluded. Instead, human studies must use somatic genomic alterations, termed ‘molecular clocks’, to trace somatic cell fates. The key principle is that the ancestry of a population of cells is revealed by the somatic alterations shared amongst the cells: closely related cells are likely to share multiple alterations, whereas distantly related cells will have few alterations in common. Thus, human lineage tracing studies rely on the notion that the clonal history of a cell is recorded in its genome. Various types of somatic genomic alterations have been exploited for lineage tracing in human tissues, including mitochondrial DNA mutations^11–24^, DNA methylation at selectively neutral loci^25–32^, allelic loss at heterozygous loci^33,34^ and single-nucleotide variants detected by genome sequencing^35–47^.

Most molecular clocks use ‘unidirectional’ measurements that count the accumulation of changes since birth to infer the relatedness of lineages. The resolution at which a molecular clock can track clonal ancestry is a function of the rate at which genomic alterations accrue. A slow rate of alteration accrual can only reveal clonal dynamics occurring over long timescales. For example, genome sequencing studies of normal skin^47^, blood^37^, intestinal crypts^38^ and endometrial glands^39^ identified multiple subclones in each tissue, but in most cases, the reconstructed lineages diverged many years in the past, and recent cell turnover was not evident in the data. In comparison, a faster rate of alteration accrual has the potential to reveal rapid and/or recent clonal dynamics, but in practice, these approaches are compromised by ‘saturation’ wherein the same pattern of alterations evolve convergently in distinct clonal populations^48^, and effectively recording stops in childhood.

In particular, somatic cell turnover is pervasive in mammalian tissues, but the dynamics of birth, death and replacement are difficult to measure. For example, in the intestine, small numbers of mitotic epithelial stem cells maintain intestinal crypts and undergo random turnover such that only one lineage persists. It takes several months in mice for one stem cell lineage to repopulate the entire crypt^1,2^. The expansion and fixation of stem cell clones presumably recur throughout life, but most fate marker methods can only record a single clonal dominance cycle.

Previous work has shown that DNA methylation at specific CpG loci can oscillate back and forth under specific conditions^49,50^, including stem cell exit from pluripotency^51,52^. In these cases, CpG oscillations occur with a period of hours to days and are therefore less useful in timing replacement dynamics that occur over months and years.

**Fig. 1:**
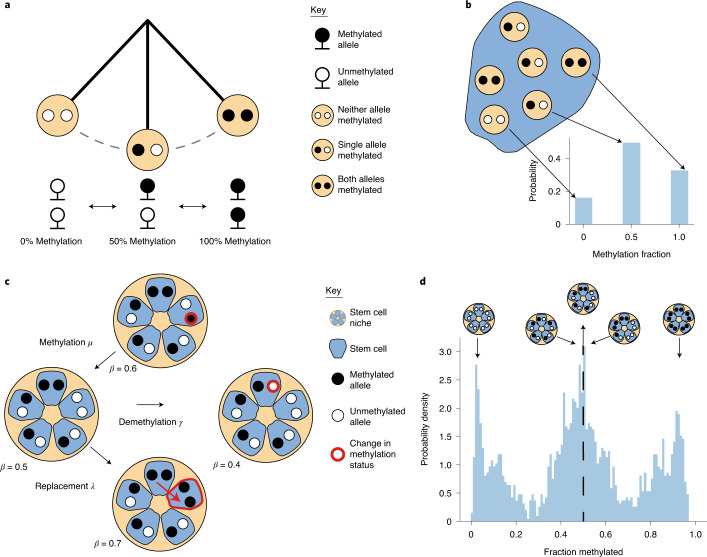
Fluctuating methylation status as a lineage tracing marker. **a**, Illustration of the three possible methylation states at a specific CpG locus within a particular cell. A cell can either be homozygously (de)methylated or heterozygously methylated at that CpG locus. It is the spontaneous transitions between these states that allow methylation to act as a lineage tracing marker. **b**, Illustration of the link between the methylation status of a given CpG locus within a particular cell and the *β* value (the fraction of methylated DNA at that locus) associated with that cell. **c**, Graphical representation of how the methylation status in a small population of five stem cells at a particular CpG locus can change over time due to (1) methylation, (2) demethylation or (3) cell replacement. **d**, Methylation (*β*) distributions from an individual crypt; the peaks near 0% and 100% correspond to a clonal methylated or unmethylated CpG locus, respectively, whereas the peak at 50% corresponds both to clonal heterozygous CpG loci and subclonal populations caught mid-sweep.

Here, we introduce the concept of FMCs, whereby epigenomic alterations reversibly change state. We test the hypothesis that certain CpG sites stochastically and measurably fluctuate in their DNA methylation levels (specifically the fraction of methylated alleles, typically referred to as the *β* value) between 0% (homozygously unmethylated CpG), 50% (heterozygous methylation) and 100% (homozygous methylation) in individual diploid cells (Fig. 1a,b). When this fluctuation occurs at a timescale on the order of decades, we show that these fCpGs can be used to infer recurrent dynamics of contemporary cell populations. Unlike traditional lineage tracing methods, which typically use a single molecular marker and thus rely on aggregating information over multiple individuals to infer the average population dynamics (exemplified in ref. ^53^), the presence of thousands of FMCs enables individual clone-specific measurements to be made. This allows inter- and intraindividual heterogeneity of the stem cell dynamics to be directly probed.

In this study, we show how fCpG methylation can be conveniently measured with commercial microarrays (Illumina EPIC arrays) that provide the methylation value at thousands of candidate fCpGs. We develop a mathematical inference methodology to extract ancestral information encoded within fluctuating sites. We validate our methodology using a simplified spatial model of a crypt cell evolution driven by different stem cell numbers then apply our fluctuating clock method to measure stem cell dynamics in individual human intestinal crypt and endometrial gland populations. The approach is further applied to whole blood to detect and distinguish between acute and chronic leukemias. The measurement of FMCs provides a powerful tool for quantifying somatic cell evolution in human tissues.

## Results

### Identification of fCpG loci

We isolated DNA from individual single colon or small intestinal crypts (31 colon samples originating from eight individuals and 28 small intestinal samples originating from seven individuals; Supplementary Table 1) and measured DNA methylation in each crypt using Illumina EPIC arrays (Methods). Samples from each tissue were treated separately to account for tissue-specific differences in DNA (de)methylation processes.

**Fig. 2:**
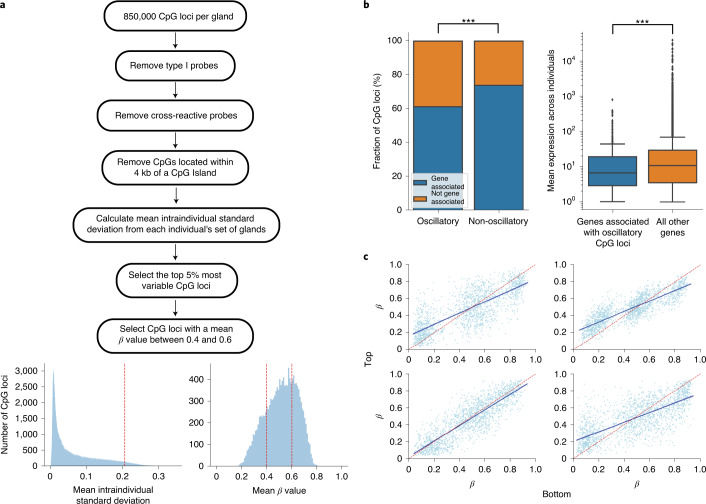
Identification of selectively neutral fCpG loci. **a**, Workflow used to identify fCpG loci that exhibit high intraindividual heterogeneity. Input data were the ~850,000 CpG loci assayed by an Illumina EPIC array. We removed type I probes and probes that cross-hybridize highly homologous DNA regions. For each CpG locus, we calculated the standard deviation for each set of approximately four crypts per individual and then calculated the mean standard deviation across the cohort as a metric for the intraindividual heterogeneity. We selected the top 5% most highly variable CpG loci and then removed CpG loci that have a mean *β* value (across the entire cohort) less than 0.4 or greater than 0.6; kb, kilobases. **b**, Left: fCpGs are enriched for CpG loci not associated with any genes (*P* = 6.5 × 10^−34^, chi-squared test). Right: the set of genes associated with fCpG loci exhibit lower average RNA expression (*P* = 6.6 × 10^−6^, two-sided Welch’s *t-*test performed following the log-transformed data) in normal colon than those genes associated with non-fCpG loci (center line, median; box limits, upper and lower quartiles; whiskers, 1.5 interquartile range). ***P < 0.001. **c**, *β* values of fCpG loci are correlated between the bottom and top halves of a crypt.

To select fCpG sites, we identified CpG loci unlikely to be actively regulated and that exhibited a high degree of intraindividual heterogeneity (Fig. 2a and Methods). This process identified 7,073 putative fCpGs within the colon sample cohort and 8,828 fCpGs within the small intestine cohort, of which 1,794 fCpGs were shared between tissue types (Supplementary Fig. 1a). There was a good correlation (*R*^2^ = 0.62) in the heterogeneity scores between colon and small intestine samples (Supplementary Fig. 1b), and fCpG loci that were exclusive to the colon had a substantially higher average variability score in the small intestine than all CpG loci (Supplementary Fig. 1c), suggesting that the relatively large number of non-overlapping loci was due to the arbitrary strictness of our identification procedure (Methods). Further analysis was performed on these shared 1,794 fCpG loci (Supplementary Table 2) to aid the generalizability of our approach. Methylation of the 1,794 fCpG loci when averaged across the 31 colon crypts was ~50%, consistent with uncorrelated stochastic (de)methylation at fCpG sites occurring independently in each crypt, but in individual crypts, we observed a characteristic trimodal ‘W-shaped’ distribution of methylation values that likely was similar to the methylation pattern of the most recent common ancestor of the crypt population (Fig. 1d).

### fCpG loci are enriched in minimally expressed genes

For CpG loci to act as a molecular clock, the loci should not be subject to strong evolutionary selection or cell-specific regulation. We compared the proportion of the 1,794 fCpG sites that were associated with a specific gene to the 428,511 CpG sites that were not identified as fluctuating (Methods). fCpG loci were strongly enriched for non-genic CpG sites (Fig. 2b; odds ratio (OR) = 1.8, chi-squared test, *P* < 0.001). We tested RNA expression using 40 normal colon samples from The Cancer Genome Atlas (TCGA)^54^ and found that the mean expression of genes associated with fCpG loci was lower than that of genes associated with the non-fCpG loci (Fig. 2b; −0.24 Cohen’s *d* calculated for log-transformed expression, Welch’s unequal variance *t*-test, *P* < 0.001). Furthermore, none of the genes that had fCpG loci in their promoter region had intermediate (10 transcripts per million (TPM)) or greater expression in single-cell RNA sequencing data of normal colon (Supplementary Fig. 2a). Together, these analyses were suggestive that methylation at the fCpG sites was unlikely to be under strict regulation or evolutionary selection in the colon.

### Methylation status of fCpGs is conserved along the crypt

Previous research has found that the methylation profile of the whole crypt is typically representative of the stem cell population at the base of the crypt^55^. To ensure that this was the case specifically for the fCpG loci identified above, we split seven crypts into their respective tops and bottoms (Supplementary Fig. 2c) and ran Illumina EPIC arrays on both halves using the same protocol described previously. Due to the low input DNA amounts, three of the samples failed the quality control step. The remaining four crypts exhibited a good correlation between the *β* values of the fCpG loci in the tops and bottoms of the crypts (Fig. 2c; *R*^2^ > 0.6, *P* < 0.001 in all cases), and binarizing the CpG calls (*β* < 0.2 encoded as 0 versus *β* > 0.8 encoded as 1) showed only 2/988 (0.2%) of fCpGs changed methylation status between crypt base and top (Supplementary Fig. 2d).

### Mathematical model of the methylation distribution of fCpGs

We hypothesized that the precise shape of the methylation *β* value distribution for fCpGs was determined by the underlying dynamics of cell evolution. To test this hypothesis in the context of intestinal crypts, we developed a mathematical model and associated Bayesian inference framework to relate the competitive dynamics of stem cells within their crypt to the measured distribution of fCpG methylation.

The mathematical model consisted of a hidden Markov model that described the time-dependent probability distribution of the number of methylated and unmethylated copies of a single CpG locus within a stem cell niche of fixed size *S*. We considered three possible processes that changed the methylation status at a given CpG locus: (1) spontaneous methylation (at constant rate *μ* per allele per stem cell per year), (2) spontaneous demethylation (constant rate *γ* per allele per stem cell per year) and (3) one stem cell replacing another stem cell (constant rate *𝜆* per stem cell per year) (Fig. 1c). We further assumed that the stem cells could be treated as a well-mixed population such that each stem cell could replace any other stem cell within the niche with equal probability. The system could be fully characterized with just two state variables: *k*, which represents the number of stem cells in the crypt with one allele methylated, and *m*, which represents the number of stem cells with both alleles methylated. By considering the possible $\left( {k,m} \right) \to \left( {k^{^\prime },m^{^\prime }} \right)$ transitions, we derived a system of ordinary differential equations describing how the probability (${{{\mathrm{P}}}}\left( {k,m|\lambda ,\mu ,\gamma ;t} \right)$) of the system being in state (*k*,*m*) changes over time (Methods and Supplementary Information). For a pool of *S* stem cells, there are 2*S* + 1 discrete states the niche methylation level could take, with a *β* value of $\frac{z}{{2S}}$ (for $z \in \left[ {0,2S} \right]$). To link the probability that a particular CpG locus has a population methylation status *z* to the output of our stem cell dynamics model, we marginalized over the various combinations of *k* and *m* that correspond to a particular *z* value, as described in the Methods.

We developed a Bayesian inference framework (Methods)^56^ that allowed for simultaneous inference of the number of stem cells (*S*), the replacement rate per stem cell (*λ*), and the methylation (*μ*) and demethylation (*γ*) rates per stem cell per allele per year for an individual gland. Thus, we could fit our model of stem cell dynamics to the data from individual crypts, allowing us to probe tissue-specific stem cell dynamics while accounting for intra- and interindividual heterogeneity.

### Stem cell dynamics are inferred with high accuracy in silico

**Fig. 3:**
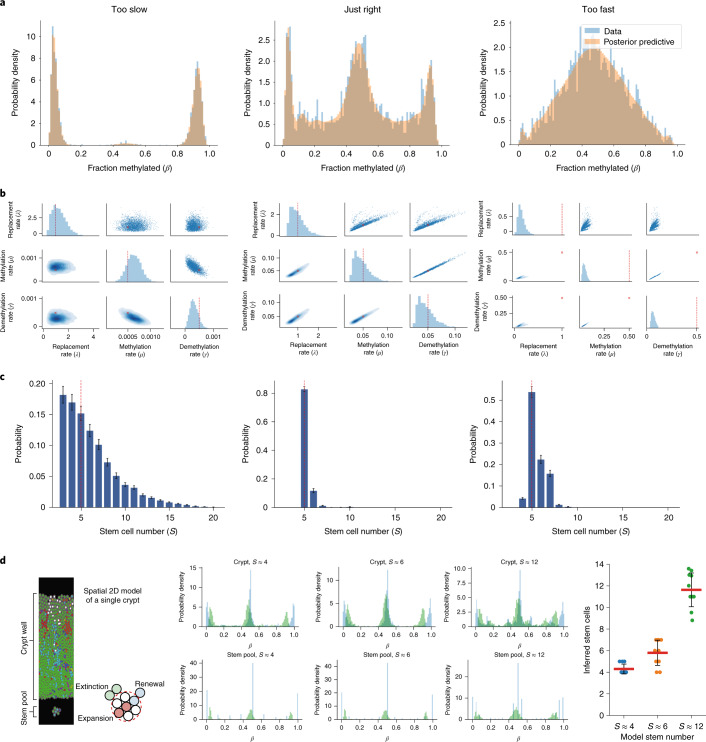
W-shaped methylation distributions are indicative of clonal dynamics. In silico evaluation of the accuracy of Bayesian inference on stem cell number (*S*), replacement rate (*λ*) and (de)methylation rates (*μ*,*γ*) as a function of input (de)methylation rates. Three regimens were evaluated: *μ* = *γ* = 0.0005 methylation events per allele per stem cell per year (‘too slow’), very high methylation rates (*μ* = *γ* = *0.5* per allele per stem cell per year (‘too fast’)) and intermediate methylation rates (*μ* = *γ*= 0.05 per allele per stem cell per year (‘just right’)). **a**, Simulated fCpG methylation distributions from individual crypts at each of three input (de)methylation rates. The characteristic W distribution is only evident for the just-right (de)methylation rate. **b**, Posterior distributions of inferred replacement and (de)methylation rates for each input (de)methylation rate. **c**, Posterior distributions of inferred stem cell number. The stem cell number posterior mean was calculated by taking the softmax of the log evidence, while the error bars were calculated from the estimated error (1 s.d.) on the log evidence. In **b** and **c**, red dashed lines indicate the true (inputted) value of the parameter. The simulated datasets each contained *S* = 5 stem cells, had a replacement rate of *λ* = 1.0 per stem cell per year, and the noise due to sampling was simulated with offsets due to background noise ${\Delta} = 0.04,{\it{\epsilon }} = 0.92$ and peak specific noise with sample size *k*_*z*_ = 100. **d**, Independent validation of the inference method on a spatial representation of the single crypt with varying stem cells. Methylation distributions are noise adjusted (Methods) for the inferences on the stem pool only. Mean inferred stem cell numbers are shown for ten replicate simulations, and the red bar represents the mean of the ten replicates while the error bars denote 1 s.d.

To verify that our Bayesian inference framework was able to accurately infer the stem cell dynamics of a crypt from FMC patterns, we generated three ‘synthetic’ crypts each containing five stem cells, a mean replacement rate of 1 per stem cell per year and a de novo (de)methylation event rate of 0.0005, 0.05 or 0.5 per allele per stem cell per year (Fig. 3a) and used our inference framework to attempt to recover the (known) underlying parameter values from the simulated methylation distributions.

At intermediate (de)methylation rates (where the clock fluctuated at a just-right rate), crypt FMC distributions showed the same characteristic W shape that we observed in the individual crypt data. Major peaks were evident near 0%, 50% and 100%, along with additional minor peaks near ~10–40% and ~60–90% which were due to recent (de)methylation events that had expanded to some, but not all, crypt cells (subclonal (de)methylation events). There are 2*S* + 1 peaks in the underlying distribution with a separation of approximately $\frac{1}{{2S}}$. Hence, the positions of these subclonal peaks hold information on the number of stem cells within the niche. Similarly, the number of fCpG loci in each peak contains information regarding the (de)methylation and replacement rates.

At low (de)methylation rates (where the clock fluctuated too slowly), the methylation distribution was essentially concentrated near 0% and 100% methylated, with a small minority of fCpG loci in the intermediate 50% methylation state, mainly due to clonal heterogeneous methylation. This is because at such a low (de)methylation rate, very few of the fCpG sites had changed their methylation status even once, and further, (de)methylation events that could distinguish a subclone were unlikely to occur. If this too slow system was to be left until it had relaxed to the steady state, the distribution would exhibit three sharp peaks near 0%, 50% and 100%, with the 50% peak containing approximately twice as many fCpG loci as the 0% or 100% peaks (due to the multiplicity of the clonal heterogeneous state).

Conversely, at high (de)methylation rates (where the FMCs fluctuated too fast) the methylation distribution approached a binomial-like distribution centered at 50%. The intuition behind this behavior is that when the (de)methylation rate was very fast, the record of clonal dynamics caused by the stem cell replacement process changing methylation allele frequency was immediately lost; hence, the system was effectively equivalent to 2*S* independent binary oscillators, with a probability of a given fCpG being in the methylated state equal to $\frac{\mu }{{\mu + \gamma }} \approx 0.5$.

Bayesian inference could not satisfactorily determine the posterior for the number of stem cells for the too slow crypt, as there were too few fCpG sites with intermediate values that held information on stem cell number. By contrast, the inference framework accurately recovered the number of stem cells for the too fast crypt, as subclonal methylation events were abundant, but the replacement rate could not be inferred accurately. This was because the clonal information that is propagated by stem cell replacement (increase/decrease in *β* values from the expanding clone) was almost immediately lost due to the high (de)methylation rate.

When the simulated (de)methylation rate was just right, the model was accurately able to recover all known parameter values with good confidence (Fig. 3b,c). We note that this in silico analysis shows that we were able to confidently confirm that the (de)methylation rate for a given set of CpG loci is within the just-right range by the presence of the characteristic W shape. Note that the range of the methylation error rates that give rise to the W shape and which are suitable for timing using our analysis is relatively broad, covering over two orders of magnitude. Despite the apparent correlations between the rate parameters, the parameters were separately identifiable within the region of the parameter space our model explores (Supplementary Information and Supplementary Fig. 7).

To further validate our Bayesian inference framework, we implemented a simplified agent-based spatial model of crypt cell evolution (Methods)^57^ where each cell (agent) incorporates molecular-level CpG tracking with (de)methylation errors possible following each cell division. We used this in silico crypt model to generate fCpG patterns from a range of stem cell pool sizes. Then applying our inference framework on the resulting CpG patterns, we were able to accurately recover the stem cell numbers (Fig. 3d) for each of the three different pool sizes (3.76 ± 0.73, 6.42 ± 0.98 and 12.39 ± 1.16 stem cells (mean ± s.d.)).

The mathematical model and inference framework relied on a number of assumptions, such as the stem cell niche being well mixed and that individual fCpG loci act independently, the impact of which was explored via generating synthetic crypts with these assumptions loosened. Our analysis was found to be generally robust to altering these assumptions (Methods and Supplementary Information).

### Measurement of stem cell dynamics in human intestine

**Fig. 4:**
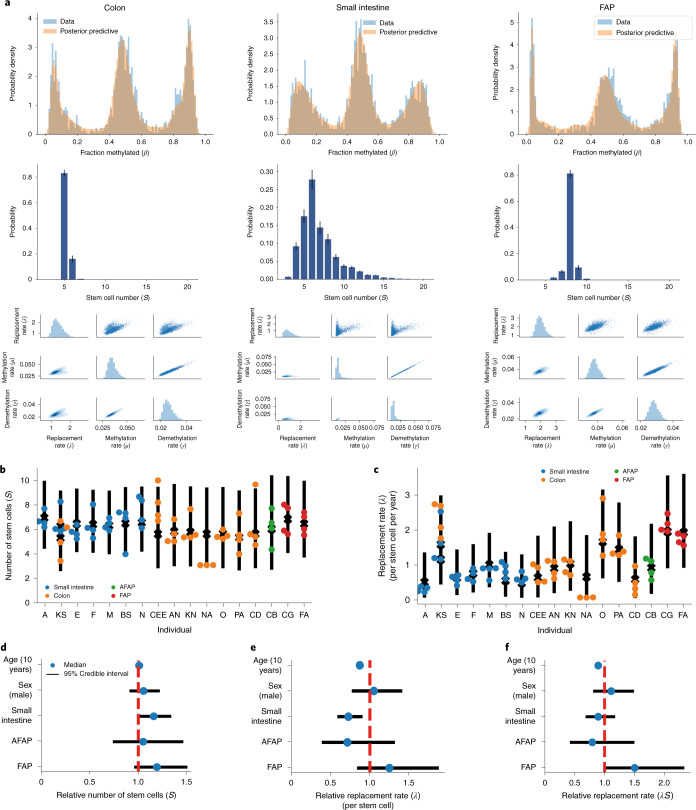
Tissue-specific differences in stem cell dynamics. The stem cell dynamics Bayesian inference framework was applied to 71 individual intestinal crypts originating from 17 individuals. **a**, Examples of the posterior predictive distributions, the discrete stem cell number posterior and the posterior for the replacement rate, methylation rate and demethylation rate in crypts derived from normal colon, small intestine and the colon of individuals with FAP (left to right). Error bars were calculated from the estimated error (1 s.d.) on the log evidence. **b**, Individual crypt and posterior mean per individual for the stem cell number; AFAP, attenuated familial adenomatous polyposis. **c**, Replacement rate per stem cell with the 95% credible range of the GLM expectation, accounting for age, sex, tissue, disease state and intra- and interindividual heterogeneity. **d**–**f**, Posterior distributions for the effect of age (per decade), sex (with female encoded as reference), tissue type and disease state on the relative number of stem cells (**d**), replacement rate per stem cell (**e**) and total number of replacements (**f**) compared to normal colon. A Bayesian parameter estimation hypothesis testing approach was taken, such that a difference was called significant if the 95% credible region did not overlap.

We measured human colon and small intestinal crypt stem cell dynamics using our FMC methodology. Methylation data were generated for each crypt individually, followed by crypt-by-crypt inference of stem cell dynamics, producing crypt-specific posterior estimates of effective stem cell number and replacement rate (Fig. 4a).

The mean number of stem cells was found to be similar across tissues, with 5.8 ± 1.7 stem cells in normal colon samples and 6.5 ± 1.0 stem cells within small intestinal glands (mean ± 1 s.d.; Fig. 4b). The replacement rate in normal colon was 1.1 ± 0.8 replacements per stem cell per year and was reduced to 0.79 ± 0.5 replacements per stem cell per year in small intestine (Fig. 4c).

We used a hierarchical Bayesian generalized linear model (GLM) to account for the hierarchical structure in our data and compared stem cell numbers and replacement rates between tissues (Methods). We found that glands from the small intestine had a greater number of stem cells (Fig. 4d; *P* < 0.05, GLM) but a lower replacement rate per stem cell than normal colon (Fig. 4e), such that the total number of replacements per crypt was not significantly different between colon and small intestine (Fig. 4f; *P* < 0.05, GLM).

Individuals with familial adenomatous polyposis (FAP) carry a heterozygous germline mutation in the *APC* gene and are at increased risk of developing colorectal cancer^58–60^. APC is a key regulator of Wnt signaling, and, therefore, pathogenic *APC* mutations cause alterations to Wnt signaling^61–63^. Wnt signaling is essential for the maintenance of intestinal stem cells^64–66^. Consequently, we hypothesized that individuals with FAP would have altered stem cell dynamics. Inference on fCpG sites in individual FAP crypts from morphologically normal colon showed that stem cell numbers were similar in FAP crypts and non-FAP colon (6.7 ± 0.3 stem cells per crypt), but the stem cell replacement rate was almost doubled at 1.9 ± 0.3 replacements per stem cell per year (Fig. 4a–c), resulting in a significantly higher total number of replacements per crypt per year in FAP (Fig. 4f).

### Stem cell dynamics in human endometrial glands

**Fig. 5:**
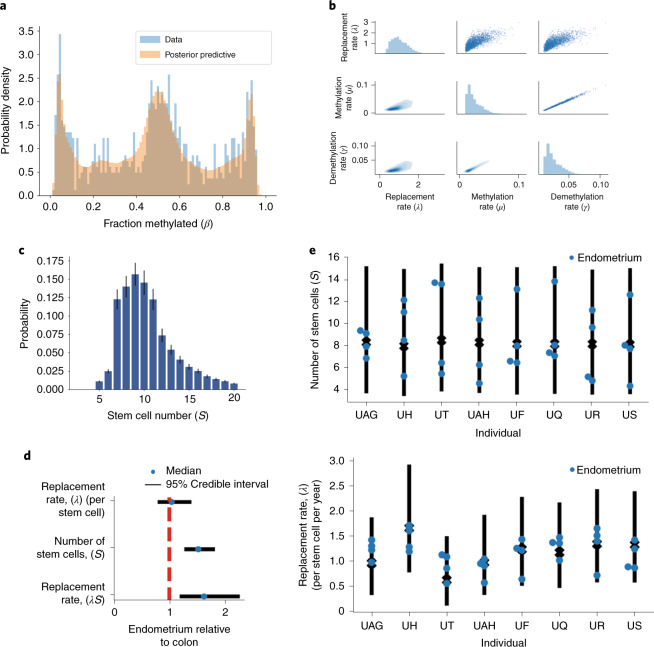
The FMC method is generalizable to other glandular tissue. The stem cell dynamics Bayesian inference framework was applied to 32 individual endometrial glands originating from eight individuals. **a**, Measured methylation *β* values (blue bars) and posterior predictive distribution (salmon overlay) are shown for an example endometrial gland. The methylation patterns exhibit a similar W shape to that observed in intestinal crypts. **b**, Posterior distributions for the replacement rate per stem cell and (de)methylation rates for the gland shown in **a**. **c**, Posterior distribution for stem cell number for the gland shown in **a**. Error bars were calculated from the estimated error (1 s.d.) on the log evidence. **d**, Inferred relative replacement rate per stem cell, number of stem cells and total replacement rate in endometrium versus colon, indicating that there are significantly more stem cells per gland in endometrium than in colon. Bars show 95% credible intervals derived from a GLM. **e**, Inferred number of stem cells and replacement rate per stem cell for each individual gland from each individual (dots) with the 95% credible range of the GLM expectation (bars).

We analyzed fCpG methylation in 32 endometrial glands derived from eight individuals using the same methodology as for intestinal crypts (Fig. 5). We derived a set of 7,721 fCpG sites, of which 807 were shared with the set of loci identified in the colon (Supplementary Table 3). The resulting methylation distributions exhibited the same characteristic W shape as in the intestine (Fig. 5a).

We then applied our Bayesian inference pipeline to each endometrial gland to infer the effective stem cell dynamics^67^. The inferred stem cell replacement rate was broadly similar compared to colon at 1.2 ± 0.3 (mean ± s.d.) replacements per stem cell per year (Fig. 5b), whereas the number of stem cells per gland was significantly higher in endometrium than in colon (*P* < 0.05, GLM), with each endometrial gland containing 8.6 ± 2.9 stem cells (Fig. 5c–e). Intriguingly, the endometrium exhibited a significantly greater degree of intraindividual variability regarding the number of stem cells (*P* < 0.05, GLM), perhaps due to the dynamic nature of the endometrium through menstrual cycles and age-related changes. We acknowledge that the stem cell structure of endometrial glands is likely more complex than that of colon crypts^68^, limiting the degree to which our simple model reflects the underlying biology. Nevertheless, the fact that we still observe large clonal peaks near 0% and 100% methylation suggests that monoclonal conversion does still occur, and our model is still applicable as a simplified caricature of the complicated dynamics present in endometrial glands.

The above analysis of human intestinal crypts and endometrial glands indicates that these small populations are maintained by small numbers of stem cells that stochastically and recurrently turnover throughout life. Experimental lineage markers can record a single clonal replacement cycle in murine crypts^1,2,69^, but FMCs continuously record ongoing stem cell dynamics that otherwise lack definitive starts or ends in adults. Although stem cell pool sizes remain constant, replacement bottlenecks and succession to a single lineage recur with a mean fixation time of 8.3 ± 5.5 years (mean ± s.d.) in the small intestine, 7.0 ± 6.4 years in the colon and 6.8 ± 4.2 years in the endometrium (Supplementary Information). Furthermore, we found that the inferred mean fixation time decreases with age, suggesting that the dynamics of stem cell replacement slow over the course of one’s lifetime (Supplementary Fig. 5b).

### FMCs in human blood

The fCpG behavior seen in intestinal crypts and endometrial glands is likely to be present across tissues. Therefore, we next searched for similar FMCs in whole human blood, which has abundant public methylation array data for normal and disease states. Unlike intestinal crypts, which recurrently drift to clonality, blood is a large, well-mixed tissue with diverse cell types and is normally polyclonal because it is produced by thousands of bone marrow stem cells^36,37^. Normal hematopoietic stem cell turnover is not synchronized. As in the intestines, CpG loci that randomly fluctuate through 0, 50 and 100% methylation in individual cells will have average methylation around 50% in normal polyclonal blood samples.

**Fig. 6:**
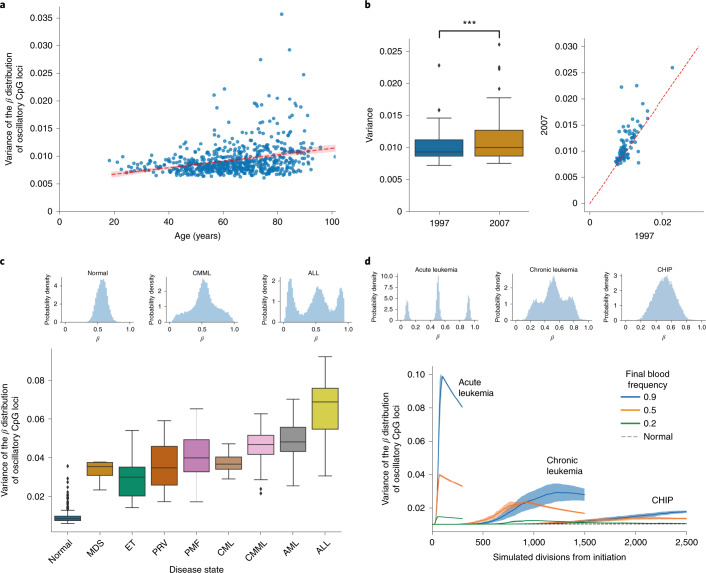
FMC dynamics can further be observed in chronic and acute leukemia. **a**, The variance of the fCpG methylation distribution experiences a gradual increase with age in healthy individuals. The confidence band was calculated via bootstrapping and represents 95% confidence intervals. **b**, Left: the variance (center line, median; box limits, upper and lower quartiles; whiskers, 1.5 interquartile range) of paired blood samples taken 10 years apart (1997 and 2007) also exhibits a small but marked increase (0.37 Cohen’s *d*, *P* = 2.8 × 10^−5^ two-sided paired *t*-test). Right: a scatterplot showing the matched variance per individual sample taken 10 years apart, demonstrating that the variance typically rises with age. ***P < 0.001. **c**, The variance of the fCpG methylation distribution (center line, median; box limits, upper and lower quartiles; whiskers, 1.5 interquartile range) is a proxy for the rapidity of the clonal expansion within the blood. In normal samples, the large stem cell population size leads to the methylation distribution being concentrated near 50% (as one would expect for uncorrelated oscillators). However, as a clonal cancerous population expands, clonal peaks begin to separate from the 50% peak. In the case of acute lymphoblastic leukemia (ALL), the large, well-separated peaks near 0% and 100% are indicative of a single clonal population making up the majority of the remaining stem cells following rapid growth; CMML, chronic myelomonocytic leukemia; MDS, myelodysplastic syndrome; ET, essential thrombocythemia; PRV, polycythemia vera; PMF, primary myelofibrosis; CML, chronic myeloid leukemia; AML, acute myeloid leukemias. **d**, Simulations confirm that a simple model of hematopoiesis can recapitulate the observed methylation distribution overserved in human data. Data represent mean ± s.e.m.; CHIP, clonal hematopoiesis of indeterminate potential.

We identified suitable fCpG loci by averaging normal whole-blood DNA methylation at ~450,000 autosomal CpG loci from a commonly used aging database of 656 healthy individuals^70^. We selected all loci (*N* = 27,634) with average values between 40% and 60% methylation in these 656 specimens. fCpGs appear to be tissue specific because only ~5% of the intestinal loci were in the blood set. Fluctuating methylation for each individual sample revealed tight distributions around 50% methylation, which can be described by its variance (Fig. 6a). Serial samples 10 years apart^71^ revealed variance to be relatively stable for an individual, with a slight significant trend for increases with age (Fig. 6b), which was also observed throughout aging (Fig. 6a).

Clonal hematopoiesis in the blood is an early step in the evolution of neoplasia and will increase variances because clonal cells will initially share the 0%, 50% and 100% methylation pattern of the progenitor. For rapid clonal expansions (that is acute leukemias), W-shaped blood distributions similar to those observed in the crypts are expected. Consistent with these expectations, whole-blood samples from different types of major hematopoietic neoplasm had higher than normal variances (Fig. 6c). ALL and AML had the highest variances and characteristic W-shaped distributions. More indolent chronic myeloproliferative or myelodysplastic whole-blood specimens showed more modest variance increases and generally lacked the W shape of the acute leukemias, crypts and glands.

### Hematopoiesis simulations

We simulated hematopoiesis to better understand how FMCs detect clonality in whole blood (Fig. 6 and Supplemental Material)^72^. Methylation fluctuates between 0, 50 and 100% in single cells, and the simulations indicate polyclonal whole-blood variance is low and stable through time because human hematopoiesis is maintained by large numbers of stem cells. Clonal expansion by a single cell synchronizes fluctuations and results in higher whole-blood variances that depend on growth rates (Fig. 6d). As in the crypts, there is a balance between clonal expansion rates, which increase population variances, and the rates at which fluctuating sites drift back to 50% average methylation, which decreases variance. A rapid expansion (less than 2 years) to high blood levels as in acute leukemias produces high variances and W-shaped distributions. The W methylation pattern resembles the methylation at 0%, 50% and 100% methylation of the initiating cell. Expansions that grow more slowly have variances greater than normal blood but lack the W shape as methylation fluctuations become increasingly desynchronized with time. These more indolent expansions are more consistent with the experimental data for chronic myeloproliferative neoplasms, which may be asymptomatic and persist for years. Clones that grow even slower and arise later, as may occur with CHIP^73^, leads to slightly higher variances, as seen with aging in the normal whole-blood cohort. A simple model with 27,634 fCpG sites and different rates of clonal expansion was broadly consistent with the experimental data from hundreds of clinical samples.

## Discussion

Here, we demonstrate how to model a class of FMCs that can reconstruct human cell population dynamics that start or recur at different times during life, using standard Illumina EPIC methylation arrays applied to bulk tissue samples. Large numbers of fCpG sites reversibly flip–flop their methylation status like an erratically swinging pendulum between 0%, 50% and 100% (representing homozygous and heterozygous (de)methylation). In polyclonal populations, these fluctuations are unsynchronized between individual cells, and the average fCpG methylation is around 50%. However, FMCs that fluctuate at a suitable fraction of the replacement rate within a clonal population leads to a characteristic W-shaped distribution with modal peaks at 0%, 50% and 100% methylation for each fCpG site following bulk measurement of the clone that resembles the state of the most recent common ancestor cell of the extant clone.

Intestinal crypts contain multiple stem cells but are clonal populations because neutral drift recurrently eliminates all stem lineages except one^1,2^. The clonality of human crypts has been previously inferred by several methods that use single or relatively few markers^22,53^. The fCpG sites represent a magnitude (>100-fold) increase in clock-like loci suitable for inferring recently occurred stem cell dynamics. These fCpG sites are common in methylation array data and show tissue specificity, likely reflecting differential gene expression between tissues (fluctuating sites are enriched at non-expressed loci). One of the major difficulties experiments with human tissue often encounter is the ‘snapshot’ nature of the data, making inference concerning dynamic processes difficult. To address this, we assessed how different temporal dynamics affect the distribution of methylation patterns across cells as measured in a ‘bulk’ sample consisting of many cells (such as an individual crypt), which, together with the relatively high de novo error rate of methylation, allowed us to track the stem cell dynamics within individual crypts. fCpG loci have different error rates, and a key to analysis is to match error rates with the underlying rate of cell dynamics. Fluctuations that occur too fast fail to record cell dynamics because fluctuating methylation becomes desynchronized even in closely related cells. Fluctuations that are too infrequent will maintain synchrony between distantly related cells and not capture more contemporary cell turnover. However, by matching FMC fluctuation rates with the biological interval of interest, we demonstrated the ability to infer stem cell dynamics within glands.

Stem cell numbers may have important fundamental roles in cancer risks because mutations that lead to cancer can only accumulate in a long-lived stem cell lineage^74^. Consistent with experiments in mice^75^, we inferred only small differences in stem cell numbers between small intestinal and colon crypts (small intestinal crypts contain approximately 16% more stem cells than colon). Whereas colon carcinoma is the fourth most common human cancer^76^, small intestinal carcinoma is between 14 and 50 times less common^76,77^, even though their tissues have similar numbers of crypts and accumulate similar numbers of mutations during aging^38^. According to the ‘bad luck hypothesis’^78^, the discrepancy in cancer rates could be explained by differences in the stem cell dynamics of the two tissues, with more stem cells dividing more rapidly carrying a higher risk of progressing to cancer. We only detect moderate differences in the number of stem cells and the replacement rates per crypt between small intestine and colon. Hence, our data and analysis indicate that much lower small intestinal carcinoma rates are unlikely to be solely attributable to the difference in stem cell dynamics between the two tissue types. We did observe a slight increase in the total number of replacements per crypt in non-dysplastic FAP colon crypts that carry heterozygous *APC* mutations, perhaps suggesting that the ‘first-hit’ loss of *APC* in the development of sporadic colorectal cancer confers a selective advantage, which may help explain why *APC* mutations are common in colorectal cancers.

We further demonstrate that fCpG dynamics are present in hematopoietic cells and can be used to reconstruct clonal dynamics within the hematopoietic system. The identity of the fCpG sites in hematopoietic cells differs from those in the epithelium, likely reflecting that fCpGs tend to be found within non-expressed genes and the fact that gene expression patterns vary between tissues. Our blood studies illustrate the ability of fCpG sites to detect clonal hematopoiesis, with increases in average fCpG variances with clonality and characteristic W-shaped distributions in acute leukemias. Chronic leukemias had intermediate fCpG variance increases and generally lacked W-shaped distributions, likely reflecting their slower growth. There was a trend for an age-related increase in fCpG variances that may reflect the increased incidence of CHIP in older people^73^.

Our stem cell dynamics inference method relies on relatively inexpensive methylation arrays, but, nevertheless, a potential limit to this technique is the requirement of high-quality DNA derived from relatively small quantities of input material. The mathematical model-based inference necessarily relies on a number of assumptions (key assumptions are discussed and evaluated), and the validity of these naturally affects the accuracy of our inference. Additionally, the dimensionality of the matrix encoding the stem cell dynamics scales quadratically with the number of stem cells; hence, our inference framework is only tractable for reasonably small numbers of stem cells.

In summary, fCpG methylation molecular clocks have many features ideal for the analysis of human cell populations. The erratic flip–flop behavior of fCpG sites is otherwise elusive in polyclonal populations but becomes detectable in clonal cell populations. FMCs can measure alterations that start or recur later in life and can infer changes that occur over a few years. Measurements are individual and gland specific, which allows us to probe intra- and interindividual heterogeneity. Large numbers of potential fCpG sites suitable for the time intervals and cell populations of interest are found on commercially available methylation arrays. FMCs enable the inference of the ongoing dynamics of many different human somatic cell populations.

## Methods

### Ethics

Tissues were collected at the University of Southern California Keck School of Medicine from excess surgical samples taken in the course of routine clinical care, with Institutional Review Board approval. Additional normal colon specimens were obtained from University College London Hospital (UCLH) Cancer Biobank (REC approval: 15/YH/0311).

### Methylation array

Crypts or endometrial glands were isolated using an EDTA washout method, as previously described^26,67^. DNA methylation was measured with EPIC bead arrays (Illumina) using the Restore protocol and the manufacturers’ protocols^79^. IDAT files were processed using the noob normalization function in the minfi R package^80^.

Blood methylation data were obtained from the Gene Expression Omnibus (GEO)^81,82^ using *β* values as provided. The datasets are GSE40279 (normal blood; Fig. 6a)^70^, GSE73115 (10-year serial samples; Fig. 6b)^83^, GSE51759 (myelodysplastic syndromes^84^), GSE42042 (essential thrombocythemia, polycythemia vera, primary myelofibrosis^85^), GSE106600 (chronic myeloid leukemia^86^), GSE105420 (chronic myelomonocytic leukemia^87^), GSE62298 (AML^88^) and GSE69229 (ALL^89^).

RNA expression data for normal tissue derived from 40 individuals were retrieved from TCGA^54^.

### Derivation of fCpG loci

To isolate those CpG sites that behave as FMCs, it was first necessary to filter out those loci that are likely to have a regulatory function or change their methylation status over the length of the crypt. This was done by selecting only those CpG sites that lie in the ‘open sea’ (further than 4 kb from a CpG island). Furthermore, probes of CpG loci that were identified^90,91^ as being cross-reactive were filtered out, along with CpG loci positioned on sex-determinant chromosomes. Given the relatively low amounts of DNA contained within a single crypt, we also filtered out probes that were likely to have experienced incomplete binding by restricting our analysis to probes that had a total intensity greater than 1,200 (arbitrary units).

The Illumina EPIC array features two different probe types, type I and type II (ref. ^91^). Type I probes feature a higher dynamic range, leading to the two probe types having different underlying distributions of *β* values. Due to difficulties in simultaneously modeling the two different probe types, and given that type I probes are overrepresented in CpG-dense regions of the genome, the analysis was restricted to type II probes.

CpG sites with fluctuating methylation were then detected by comparing between-individual to within-individual heterogeneity in methylation value. At fluctuating sites, we expect the average methylation in non-clonal bulk samples to follow a distribution centered on 0.5 (because methylation at the site is uncorrelated between the multiple lineages that make up the bulk sample), whereas in individual clonal samples, the methylation value can take any value between 0 and 1. Thus, to select for fCpG loci, we selected CpG sites that had the highest 5% of variance in *β* value between individual samples and then filtered these for sites with mean methylation across all samples and individuals of ~0.5 (mean *β* value between 0.4 and 0.6) (Fig. 2a).

To demonstrate technical accuracy in methylation measurement from the small amounts of DNA in single small intestinal crypts (~400 cells), colon crypts (~2,000 cells) or endometrial glands (~5,000 cells), we identified similar fCpG sites on the X chromosome and compared methylation between male and female individuals. In males, there is only a single copy of the X chromosome; hence, only two modal peaks near 0% and 100% methylation should be present in clonal populations, as opposed to the trimodal distribution observed on autosomes. Consistent with the ability to measure fluctuating methylation in small tissue samples, the X chromosome fluctuating sites exhibited trimodal W-shaped distributions in female colon crypts and bimodal ‘U-shaped’ distributions in male colon crypts (Supplementary Fig. 1d). This observation is supportive of the hypothesis that the methylation distribution of fCpG loci is reflective of that of the most recent recurrent clone rather than varying with cell type or differentiation status.

We compared methylation of fluctuating sites between crypts from the same individual. If fluctuating methylation occurs stochastically and without biological regulation, then each crypt should independently evolve a unique pattern of fCpG site methylation. Intercrypt comparison between crypts within the colon or small intestine, both across the set of crypts sampled from each individual and across crypts from different individuals, showed that fluctuating methylation patterns between crypts were uncorrelated (Supplementary Fig. 2e). There was weak correlation of fluctuating methylation patterns between crypts for younger individuals (age <30 years), but this was lost with advancing age (Supplementary Fig. 2f).

### Mathematical model of methylation within the stem cell niche

We developed a stochastic model to describe how the fraction of methylated alleles (*β* value) in the stem cell niche of a given CpG locus changes over time. This model draws on previous attempts^1,75^ to model the behavior of the stem cell niche in colonic crypts but with a number of modifications that account for the differences when considering methylation as a lineage tracing marker rather than DNA. Namely, while DNA mutations occur relatively infrequently, allowing for a model that only considers a single mutant population expanding or contracting with reference to a single wild-type population, the relatively high methylation switching rate requires us to consider the potential of multiple clones existing simultaneously. Further, while DNA mutations can be generally regarded as irreversible, the methylation status of a given cell (that is, whether a particular cell is homozygously (de)methylated or heterozygously methylated) can theoretically flip–flop, necessitating a careful consideration regarding the possible ways the overall *β* value can change.

For this reason, we made the simplifying assumption that the population was well mixed, such that any of the *S* stem cells can replace any of the other *S* – 1 stem cells with equal probability and that these replacements occur at a constant rate *λ* per stem cell. This assumption greatly simplified our analysis, as the system can be fully characterized using just two state variables: *k* – the number of stem cells containing a single methylated allele, and *m* – the number of stem cells containing two methylated alleles. The admitted states are constrained by the inequality $0 \le k + m \le S$ for a total of $\frac{1}{2}\left( {S + 1} \right)\left( {S + 2} \right)$ states.

Along with the replacement process, we assumed that a previously unmethylated CpG locus could spontaneously become methylated with a rate *μ* per year and, conversely, that a previously methylated CpG locus could spontaneously become demethylated with a rate *γ* per year.

To develop the series of ordinary differential equations that fully determine the system, we considered the ways in which a state (*k*,*m*) could transition to a state $\left( {k^\prime ,m^\prime } \right)$. As an example, if we consider Fig. 1c, we observe that of the *S* = 5 stem cells, 3 of the stem cells are heterozygously methylated, and 1 of the cells is homozygously methylated; hence, the system is initially in state (*k* = 3,*m* = 1). To transition to state $\left( {k^\prime = 3,m^\prime = 2} \right)$, the homozygously methylated stem cell must clonally expand, replacing the homozygously demethylated cell. The rate at which any one of the stem cells replaces another is *λS* = 5*λ*, but of the *S*(*S* – 1) = 20 possible transitions, only 1 would lead to the desired (3,2) state; hence, the rate at which the system transitions $\left( {3,1} \right) \to \left( {3,2} \right)$ is $\frac{1}{{20}} \ast 5\lambda = \frac{1}{4}\lambda$. We continue this process (Supplementary Information) considering the general transition $\left( {k,m} \right) \to \left( {k^\prime ,m^\prime } \right)$, deriving the following master equation:

$$\begin{array}{l}\frac{{d{{{\mathrm{P}}}}\left( {k,m|\lambda ,\mu ,\gamma ;t} \right)}}{{dt}} = \left( {S - m - \left( {k - 1} \right)} \right)\left( {\left( {k - 1} \right)\frac{\lambda }{{S - 1}} + 2\mu } \right){{{\mathrm{P}}}}\left( {k - 1,m|\lambda ,\mu ,\gamma ;t} \right)\\ + \left( {m - 1} \right)\left( {S - \left( {m - 1} \right) - k} \right)\frac{\lambda }{{S - 1}}{{{\mathrm{P}}}}\left( {k,m - 1|\lambda ,\mu ,\gamma ;t} \right)\\ + \left( {k + 1} \right)\left( {\left( {m - 1} \right)\frac{\lambda }{{S - 1}} + \mu } \right){{{\mathrm{P}}}}\left( {k + 1,m - 1|\lambda ,\mu ,\gamma ;t} \right)\\ + \left( {k + 1} \right)\left( {\left( {S - m - \left( {k + 1} \right)} \right)\frac{\lambda }{{S - 1}} + \gamma } \right){{{\mathrm{P}}}}\left( {k + 1,m|\lambda ,\mu ,\gamma ;t} \right)\\ + \left( {m + 1} \right)\left( {S - \left( {m + 1} \right) - k} \right)\frac{\lambda }{{S - 1}}{{{\mathrm{P}}}}\left( {k,m + 1|\lambda ,\mu ,\gamma ;t} \right)\\ + \left( {m + 1} \right)\left( {\left( {k - 1} \right)\frac{\lambda }{{S - 1}} + 2\gamma } \right){{{\mathrm{P}}}}\left( {k - 1,m + 1|\lambda ,\mu ,\gamma ;t} \right)\\ - \left( {2\left( {k\left( {S - k} \right) + m\left( {S - k - m} \right)} \right)\frac{\lambda }{{S - 1}} + \left( {2S - \left( {k + 2m} \right)} \right)\mu + \left( {k + 2m} \right)\gamma } \right)\\{{{\mathrm{P}}}}\left( {k,m|\lambda ,\mu ,\gamma ;t} \right)\end{array}$$

This linear series of differential equations can be solved computationally by rewriting the equations into a matrix equation, $\frac{{d\mathop{P}\limits^{\rightharpoonup} \left( t \right)}}{{dt}} = {\it{T}}\mathop{P}\limits^{\rightharpoonup} \left( t \right)$ and applying matrix exponentiation to the resulting transition matrix *T*.

$$\mathop{P}\limits^{\rightharpoonup} \left( t \right) = e^{t{\it{T}}}\mathop{P}\limits^{\rightharpoonup} \left( {t = 0} \right)$$

During the very early stages of embryogenesis, the existing methylation patterns inherited from parental gametes are largely erased before a large wave of de novo methylation remodels the entire genome, resulting in a bimodal methylation distribution^92^. Given that all the stem cells within a niche are initially clonal, we thus assumed that it was equally likely to find a given fCpG locus as homozygously methylated or unmethylated across all the stem cells within the niche at time 0. Further study is necessary to ensure the validity of this assumption.

$${{{\mathrm{P}}}}\left( {k,m|\lambda ,{\it{\upmu }},\gamma ;t = 0} \right) = \left\{ {\begin{array}{*{20}{c}} {0.5} & {{\textrm{if}}} & k & = & {0 \wedge m} & = & S \\ {0.5} & {{\textrm{if}}} & k & = & {0 \wedge m} & = & 0 \\ 0 & {{\textrm{otherwise}}} & {} & {} & {} & {} & {} \end{array}} \right.$$

However, the methylation status of individual cells is not available using methylation arrays; hence, the hidden states must be marginalized over to calculate the probability of there being *z* methylated copies within the stem cell niche (note that $0 \le z \le 2S$). This can be achieved by summing the various combinations of *k* and *m* states that satisfy the equation *z* = *k* + 2*m*.

$${{{\mathrm{P}}}}\left( {z|\lambda ,\mu ,\gamma ;t} \right) = \mathop {\sum }\limits_{m = 0}^S \mathop {\sum }\limits_{k = 0}^{S - m} {{{\mathrm{P}}}}\left( {k,m|\lambda ,\mu ,\gamma ;t} \right)\delta _{k + 2m,z}$$

The resulting distribution of ${{{\mathrm{P}}}}\left( {z|\lambda ,\mu ,\gamma ;t} \right)$ can qualitatively reproduce the characteristic W shape exhibited in the methylation fraction of individual crypts.

### Error model

The probability distribution calculated above, ${{{\mathrm{P}}}}\left( {z|\lambda ,\mu ,\gamma ;t} \right)$, gives the probability that exactly *z* of the 2*S* alleles (across *S* stem cells) are methylated at a particular CpG locus; however, the Illumina EPIC array quantifies the methylation level at specific loci aggregated over the whole crypt. As such, we introduced an error model to link the measured *β* value with the ‘true’ *z* value at a specific site. We chose to model the measured *β* values as a mixture of *z* *β*-distributed random variables, each with a mean value determined by *z* and a scale parameter *k*_*z*_.

To account for the background noise of the array, the mean value of each *β* peak was set to be equal to a linear transform of *z*: $x = \left( {{\it{\epsilon }} - {{{\mathrm{{\Delta}}}}}} \right)\frac{z}{{2S}} + {{{\mathrm{{\Delta}}}}}$, with the parameters describing this transform (${\epsilon }$ and Δ) to be inferred. The scale parameters (sometimes referred to as the sample size), $\vec \kappa$, of each *β* peak were modeled as hierarchical, with each *κ*_*z*_ being drawn from a lognormal distribution parameterized in terms of the population mean, *θ*, and its standard deviation, *σ*. These hyperparameters were also inferred during the Bayesian inference.

### Likelihood and prior functions

As rate parameters are naturally positive quantities, *λ*, *μ* and *γ* were constrained to positive real values by defining the prior distributions in terms of positive half-normals with a scale informed by prior literature. Following the finding of Nicholson et al.^53^ that the replacement rate is approximately 1.3 replacements per stem cell per year, we set the scale of the prior on the replacement rate equal to 1. Similarly, *θ* and *σ* were also constrained to positive values using broad half-normal prior distributions, with a scale of 500 and 50, respectively. Previous work has found that methylation fidelity can vary dramatically across the genome, from ~10^−4^ to 10^−2^, and we will take an estimate of 10^−3^ per division as a reasonable scale^93^. If we assume intestinal stem cells divide every ~3 d and we consider that our definition of *μ*, *γ* is in units of (per allele per year), this corresponds to a (de)methylation rate of ~0.05. We note that the inference is relatively insensitive to the exact choice of prior on the (de)methylation rate (Supplementary Fig. 7b,c). The lognormal hierarchical prior distribution naturally constrains $\vec \kappa$ to real values. The ‘offsets’ in the linear transform, Δ and ${\it{\epsilon }}$, were constrained to lie between 0 and 1 by placing a *β* distribution on each parameter, such that the mean prior value was 0.05 and 0.95, respectively.

The behavior of individual CpG loci was assumed to be independent, such that the likelihood of all *N* = 1,794 CpG loci was simply the product of the per-CpG likelihood computed according to the mathematical model outlined above.

Likelihood:

$$x = \left( {{\it{\epsilon }} - {{{\mathrm{{\Delta}}}}}} \right)\frac{z}{{2S}} + {{{\mathrm{{\Delta}}}}}$$

$${{{\mathrm{P}}}}\left( {\beta _i|z,{{{\mathrm{{\Delta}}}}},{\it{\epsilon }},\kappa _z} \right) = \frac{{\beta _i^{\kappa _zx - 1}\left( {1 - \beta _i} \right)^{\kappa _z\left( {1 - x} \right) - 1}}}{{{{{\mathrm{B}}}}\left( {\kappa _zx,\kappa _z\left( {1 - x} \right)} \right)}}$$

$${{{\mathcal{L}}}}\left( {\lambda ,\mu ,\gamma ,{{{\mathrm{{\Delta}}}}},{\it{\epsilon }},\vec \kappa ,S|\vec \beta } \right) = \mathop {\prod }\limits_{i = 1}^N \mathop {\sum }\limits_{z = 0}^{2S} {{{\mathrm{P}}}}\left( {\beta _i|z,{{{\mathrm{{\Delta}}}}},{\it{\epsilon }},\kappa _z} \right){{{\mathrm{P}}}}\left( {z|\lambda ,\mu ,\gamma ;t} \right)$$

Hyperpriors:

$$\theta \sim {{{\mathrm{halfnormal}}}}\left( {500} \right)$$

$$\sigma \sim {{{\mathrm{halfnormal}}}}\left( {50} \right)$$

Priors:

$$\lambda \sim {{{\mathrm{halfnormal}}}}\left( {1.0} \right)$$

$$\mu \sim {{{\mathrm{halfnormal}}}}\left( {0.05} \right)$$

$$\gamma \sim {{{\mathrm{halfnormal}}}}\left( {0.05} \right)$$

$${{{\mathrm{{\Delta}}}}}\sim {{{\mathrm{\beta}}}}\left( {5,95} \right)$$

$${\it{\epsilon }}\sim {{{\mathrm{\beta}}}}\left( {95,5} \right)$$

$$\kappa _z\sim {{{\mathrm{lognormal}}}}\left( {\ln \left( {\frac{{\theta ^2}}{{\sqrt {\theta ^2 + \sigma ^2} }}} \right),\sqrt {\ln \left( {1 + \frac{{\sigma ^2}}{{\theta ^2}}} \right)} } \right)$$

### Bayesian inference

A Bayesian inference methodology was developed to infer the biological model parameters (number of stem cells within the stem cell niche (*S*), replacement rate per stem cell per year (*λ*), and methylation (*μ*) and demethylation (*γ*) rate per CpG locus per stem cell per year) directly from the distribution of FMC *β* values for each crypt.

Investigation of simulated datasets revealed that the resulting posterior distributions were multimodal, with each *S* value associated with a local maxima (due to the correlation in the posterior between *S* and *λ*). This multimodality can make the posterior difficult to explore using traditional Markov chain Monte Carlo techniques, such as Hamiltonian Monte Carlo. To overcome this, a nested sampling method^94^ was developed to calculate the Bayesian evidence (marginal probability density, ${{{\mathcal{Z}}}}$) of each *S* value considered ($S \in \left[ {3..20} \right]$) while simultaneously generating samples from the posterior associated with each value of *S*. The probability of *S* for a given crypt can then be calculated as

$${{{\mathrm{P}}}}\left( {S|\vec \beta } \right) = \frac{{{{{\mathcal{Z}}}}\left( {S|\vec \beta } \right)}}{{\mathop {\sum }\nolimits_j {{{\mathcal{Z}}}}\left( {S_j|\vec \beta } \right)}}$$

The full posterior can be approximated by drawing samples from each *S* mode with a weight equal to the inferred probability of *S*. The nested sampling was performed using dynesty^95^, a Python implementation of the nested sampling algorithm, using the ‘rwalk’ sampling option, such that new live points are generated from existing live points under random walk behavior.

To ensure that the posterior samples had converged to the equilibrium distribution, four independent samples were run with random initializations for each sample, and the rank-normalized potential scale reduction statistic ($\hat R$) was calculated^96,97^. $\hat R$ was found to be less than 1.1 (a typical threshold used to determine convergence) in all cases. The inference code can be obtained from https://github.com/CalumGabbutt/flipflop.git (ref. ^56^).

### Impact of simplifying assumptions

Our mathematical model of intestinal stem cell niche dynamics inevitably rested on a number of simplifying assumptions. We investigated the impact of these assumptions.

First, we assumed a well-mixed population. This differed from previous prominent modeling approaches, foremost the approach of Lopez-Garcia et al.^1^ who assumed that stem cells were organized in a ring geometry where replacement could only happen between neighboring cells on the ring (Supplementary Fig. 3a and Supplementary Information). We used stochastic simulation to explore the effect of a well-mixed versus ring geometry. Across biologically relevant numbers of stem cells ($S \lesssim 10$), the differing geometry was found to have a negligible effect on the resulting fCpG methylation distribution (Supplementary Fig. 3b). We performed statistical inference upon these simulations (using the inference framework that makes the well-mixed assumption) and were able to accurately recover the known parameters (Supplementary Fig. 3c). We note that Lopez-Garcia et al.’s model only needed to consider the clonal expansion or retraction of a single labeled clone, whereas our model had to account for the possibility of multiple labeled clones due to the increased mutation rate of the epigenome; hence, the well-mixed assumption was chosen to minimize mathematical complexity. Further, we note that live-imaging data from mouse crypts^69^ show that murine stem cells can exchange places within the niche, suggesting that the stem cell population may be neither strictly ring-like nor well mixed but rather a hybrid model between the two extremes.

Second, we neglected genetic ‘linkage’ between different CpG loci (each cell carries a set of linked CpGs) to prevent mathematical complexity. We explored the effect of linkage using the same well-mixed Gillespie simulations as above and found that the mean methylation per peak of the individual crypts simulated with linkage exactly matches that of the analytic probability distribution that we derived but that the individual crypts exhibit a greater degree of variability than that predicted by sampling from the analytic model (Supplementary Fig. 6). Consequently, credible intervals of the posterior inferred with our non-linkage inference method will be marginally too narrow.

Third, we assumed that all of the fCpG loci that we had identified as fluctuating were not under selection or active regulation. We explored the consequence of a fraction of CpG sites not behaving in a fluctuating manner on the accuracy of the inference (Supplementary Information and Supplementary Fig. 4). Including non-fluctuating sites caused a systematic underestimation of the replacement rate, but when the number of non-fluctuating sites was sufficiently low (≲5%), the number of stem cells and the replacement rate could still be accurately inferred.

Finally, we assumed that the replacement rate, methylation rate and demethylation rate are constant over an individual’s lifetime. While previous research suggests that the stem cell division rate lowers over an individual’s lifetime^98^, and our findings are consistent with such a decrease, it is likely that both the replacement rate and the methylation error rate are proportional to the cell division rate, such that the ratio of the two rates does not change over time. In this way, our model describes the stem cell dynamics of an individual crypt averaged over an individual’s lifetime.

### Tissue-specific differences in stem cell dynamics

To compare the stem cell dynamics of different tissue and disease types in a statistically rigorous manner, we must account for the hierarchical individual structure (that is, we have multiple glands from each individual that are likely to be correlated) while controlling for the age and sex of each individual. We developed a hierarchical Bayesian GLM using a log-link function to constrain our dependent variable to be positive (presented fully in the Supplementary Information) and take a hypothesis testing by parameter estimation approach (that is, the difference between small intestine and colon is statistically significant if the 95% equal-tailed credible interval excludes 0).

### Spatial model of the crypt

A crypt ignoring villi in the small intestine forms a cylindrical geometry with stem cells at the base and a crypt wall moving up the crypt. Here, we have developed an off-lattice mechanistic agent-based model of the human crypt using the hybrid automata library (HAL) modeling framework^99^ capable of representing a crypt of the small intestine or colon (Fig. 3d). The cylindrical unit is separated into two compartments, the stem cell compartment represented as a pool at the base of the crypt and then the wall of the crypt where transit amplifying cells are pushed upward until they are removed from the top of the crypt. The spatial model of the crypt is dynamic in the sense that the *x* and *y* dimensions are calculated using the total populations size (*N*_*T*_) and the stem cell pool radius (*ψ*). The *x* dimension is defined as *x* = 2*πψ*. The center of the stem cell pool is placed such that the origin of the center of this circular stem cell pool whose size, and thus number of stem cells allowed within this pool, is placed at (*h*,*k*) where *h* = *x*/2 and *k* = *ψ* + 5 Division for each stem cell is defined by *ρ*_*c*_, which is randomly assigned as the hourly cell cycle defined by $p_c\sim U\left( {\rho _{min},p_{max}} \right)$, where *ρ*_*min*_ and *ρ*_*max*_ are *ρ* ± 4 h.

As a cell approaches *ρ*_*c*_, the cells diameter doubles for the 5 h/time steps preceding the cell’s division. Following division, both daughter cells occupy this space. When a stem cell (defined by $d\left( {x_c,y_c} \right) \le \psi$ where $d\left( {x_c,y_c} \right) = \sqrt {\left( {x_c - h} \right)^2 + \left( {y_c - k} \right)^2}$) divides, the daughter cells can be placed in any arrangement around the parent cell’s *x*_*c*_ and *y*_*c*_ position; differentiated cells can only be placed vertically (that is, the *x*_*c*_ values are equal). The base of the crypt wall is set just above the origin of the stem cell pool plus *ψ* and a small offset to provide space so that no cell forces interact between the stem cell pool and the base of a stem cell wall. If $d\left( {x_c,y_c} \right) > \psi$, then the cell is moved to the base of the stem cell wall where the cell’s new position (*x*_2_,*y*_2_) is given as *y*_2_, and *x*_2_ is given by the cell’s exit radians, *rad*_*s*_, given by $atan2\left( {y_c,x_c} \right)$ so that the cells position along the *x* dimension is $x_2 = \left( {rad_s + \pi } \right)\left( {\frac{x}{{2\pi }}} \right)$. Boundary conditions for the cells within the crypt wall are periodic (that is, allowed to wrap around) and no flux at the top and bottom of the crypt (that is, no cell can breach these boundaries). A run step in the model is hourly, and updates to cell positions occur for the whole crypt and are applied at each time step. We give each cell 1,794 CpG loci (with the possible status of 0 for demethylated or 1 for methylated). At each division, these loci can switch methylation status at a rate defined by *ω* following division.

At each hourly time step, we assume that the forces acting on each individual cell are at equilibrium, $F_{c_i} = 0$, where $F_{c_i}$ is equal to the contact force between cell *i* and its neighbors. For two cells whose radii are *R*_*i*_ and *R*_*j*_, respectively, the contact force between them is based on a linear spring constant model (Hooke’s law) and is calculated as

$$F_{c_{ij}} = \left\{ {\begin{array}{*{20}{c}} {k_i\frac{{{{{\mathrm{{\Delta}}}}}R_{ij}}}{{R_i + R_j}}} & {if} & {\frac{{{{{\mathrm{{\Delta}}}}}R_{ij}}}{{R_i + R_j}}} & > & 0 \\ 0 & {if} & {\frac{{{{{\mathrm{{\Delta}}}}}R_{ij}}}{{R_i + R_j}}} & < & 0 \end{array}} \right.$$

Assuming that each cell has the same spring constant *k*, the overlap of cells ($\frac{{{{{\mathrm{{\Delta}}}}}R_{ij}}}{{R_i + R_j}}$) and the overall number of cells in contact with any given cell (*n*_*i*_) give the velocity for an individual cell, $v_i = k\mathop {\sum }\nolimits_{j = 1}^{n_j} \frac{{{{{\mathrm{{\Delta}}}}}R_{ij}}}{{R_i + R_j}}$. The modeling framework can be obtained from https://github.com/MathOnco/flipflopspatialmodel.git (ref. ^57^).

### Inference of stem cell numbers on the spatial model

To provide insights into the FMC signal from a first principles model of the homeostatic crypt (balanced birth/death with a methylation error rate), we have to add noise to the output data of the spatial model. This is because the inference framework is designed to fit the noisy experimental data and that fCpG sites with values of zero or one are not captured within the data. To add a small amount of noise to the output of the perfect methylation distribution’s output by the spatial model, a binomial is used with two offsets to provide a distribution that the inferences can be performed on. For each *β* value, a sample size (*κ*) of 1,000 is taken from a *β* distribution using an offset from 0 (Δ = 0.04) and an offset from 1 (${\it{\epsilon }} = 0.92$) (Fig. 3d). The script required to add noise to this model is accompanied with the inference framework (see add_noise.py). Once the *β* values with noise are added, the inference framework is executed for each model simulation’s *β* value distributions for across stem cell number ranges from 2 to 9, 3 to 10 and 8 to 15, respectively, using 400 live points for the dynesty sampler^95^.

### Reporting Summary

Further information on research design is available in the Nature Research Reporting Summary linked to this article.

## Online content

Any methods, additional references, Nature Research reporting summaries, source data, extended data, supplementary information, acknowledgements, peer review information; details of author contributions and competing interests; and statements of data and code availability are available at 10.1038/s41587-021-01109-w.

## Supplementary information

Supplementary Information Supplementary Figs. 1–7, Discussion and Tables 1–3.

Reporting Summary.

Supplementary Table 1 Sample information, including individual ID, age and sex, the tissue the sample derived from, any underlying diseases and whether the individual was diagnosed with cancer.

Supplementary Table 2 The fCpG loci identified and the *β* values of the intestinal samples.

Supplementary Table 3 The fCpG loci identified and the *β* values of the endometrial samples.

## Data Availability

Illumina EPIC array data (colon, small intestine and endometrium) collected in the process of this study are currently available at the European Genome–Phenome Archive (EGA) (accession EGAS00001005514). Figs. 2, 4 and 5 are associated with these data. Sample information is presented in Supplementary Table 1. The fCpG loci identified and the *β* values of intestinal and endometrial samples are presented in Supplementary Tables 2 and 3, respectively.

## Code availability

The Bayesian inference framework to infer the stem cell dynamics of individual crypts from the distribution of *β* values of fCpG loci can be obtained on GitHub at https://github.com/CalumGabbutt/flipflop.git (ref. ^56^). The agent-based spatial modeling framework of the crypt is available at https://github.com/MathOnco/flipflopspatialmodel.git (ref. ^57^).

The blood simulations illustrating how the methylation distribution changes following a rapid clonal expansion can be obtained (along with sample simulation results) at https://github.com/MathOnco/flipflopblood.git (ref. ^72^). A graphical user interface compatible with most operating systems is accompanied to allow for rapid evaluation of different parameters.
