# Supplementary material for: Fluctuating methylation clocks for cell lineage tracing at high temporal resolution in human tissues
Source: Nat Biotechnol. 2022 Jan 3;40(5):720–30. doi: 10.1038/s41587-021-01109-w (PMC9110299; doi:10.1038/s41587-021-01109-w)
Supplement: Supplementary file 1 — Supplementary Figs. 1–7, Discussion and Tables 1–3. [file 41587_2021_1109_MOESM1_ESM.pdf]

nature portfolio

https://doi.org/10.1038/s41587-021-01109-w

Supplementary information

Fluctuating methylation clocks for cell lineage tracing at high temporal resolution in human tissues

In the format provided by the authors and unedited

Supplementary Information

Supplementary Figures

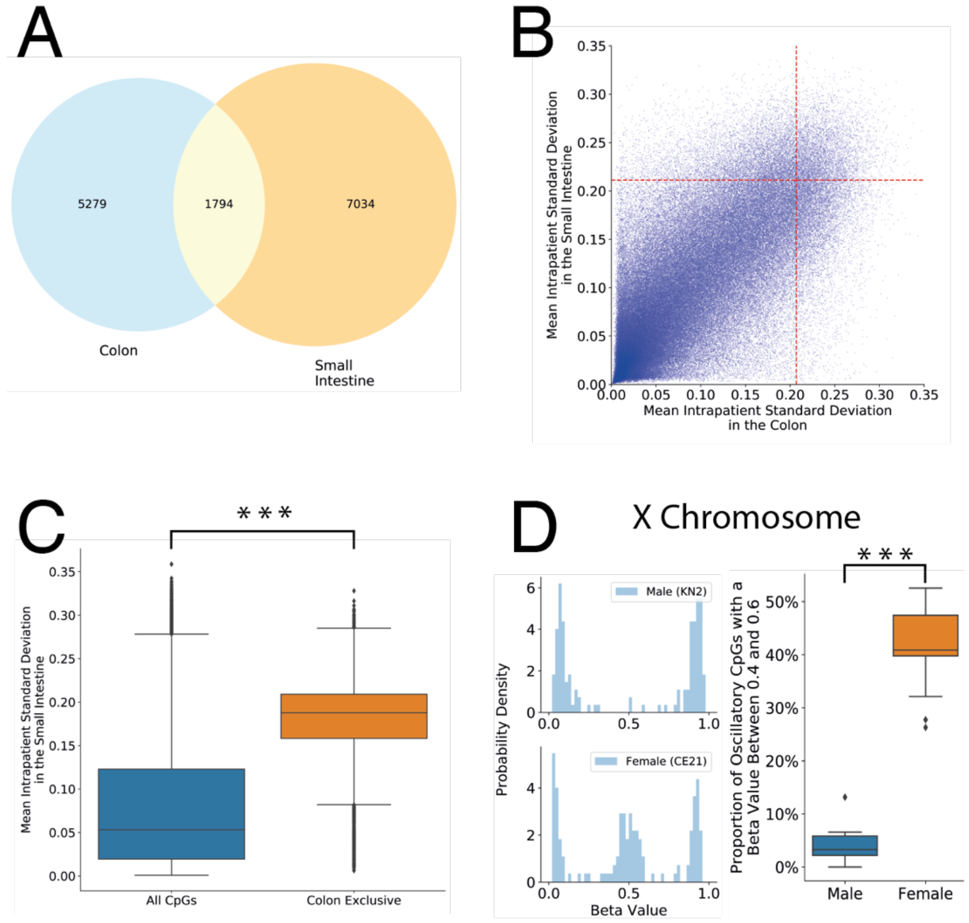

Supplementary Figure 1: Additional analysis of fCpG identification process

A: Venn diagram showing the overlap of CpG loci identified as fCpGs in the colon and the small intestine. **B:** Scatter density plot (with the density plotted on the log-scale) of the heterogeneity metric (mean intra-patient standard deviation) of CpGs in the colon and small intestine, with the cutoff of the top 5% most heterogenous loci indicated in red. **C:** Comparison of the heterogeneity metric of the colon exclusive fCpG loci (i.e. those identified in the colon but not the small intestine) to all type II CpGs, within the small intestine samples (center line, median; box limits, upper and lower quartiles; whiskers, 1.5 IQR). The colon exclusive CpG loci are significantly more variable ($p < 2 \times 10^{-16}$, two-sided Mann Whitney U test). **D:** An extension of the fCpG identification process to CpG loci located on the X chromosome. (left) We present example methylation distributions for these X-chromosome fCpG loci for a male and a female crypt, confirming the predictions from theory that the male crypts lack the peak at 50% as they contain only a single copy. (right) To test whether this relationship holds in general, we compare the proportion of fCpG's on the X chromosome with an intermediate beta value ($0.4 \leq \beta \leq 0.6$) between all colon crypts from males and females (center line, median; box limits, upper and lower quartiles; whiskers, 1.5 IQR), confirming that males have a significantly lower probability mass near 50% ($p = 3.9 \times 10^{-6}$, two-sided Mann Whitney U test).

Evidence for fluctuating human CpG sites

Oscillatory DNA methylation has been previously documented in cell lines systems where changes in DNA methylation have been directly observed and correlated with changes in gene expression and developmental cell fates[1–4]. These active changes in DNA methylation are modulated by Tet and Dnmt family enzymes, and occur over hours or a few days. By contrast, the erratic fluctuations at fCpG loci outlined in this paper occur over years and in adult human tissues. For these fCpG loci, the proposed mechanism is more likely stochastic replication errors, where DNA methylation and demethylation are equally likely.

Such stochastic, neutral changes in DNA methylation are more likely to occur in non-genic or non-regulatory CpG sites rather than in expressed genes that could alter cell phenotypes. The identified fCpG loci were relatively enriched for non-genic CpG sites (Fig. 2B) and were about three times less likely to be in promoter regions that largely control gene expression. Moreover, genes with promoter fCpG sites had significantly lower expression than genes without promoter fCpG loci ($p < 0.001$ Welch's t-test performed upon the log-transformed data, Fig. S2A). We note that none of the genes with promoter fCpG sites had an expression greater than 10 TPM, a typical cutoff for “intermediate” expression. Hence, fCpG methylation variation is unlikely to be associated with cell differentiation or actively confer significant changes in phenotype.

Although direct serial observations of the methylation and demethylation of fCpG loci in the same human colon crypt are impractical, the proposed stochastic mechanism has testable predictions. We illustrate some of these predictions by comparing methylation extremes, defined as “0” if the methylation is less than 0.2 and “1” if the methylation is greater than 0.8 (Fig. S2B). The clonal crypts have hundreds of fCpG sites with values of either “0” or “1”, which should effectively serve as lineage barcodes over short time intervals. (CpG sites with methylation between 0.2 and 0.8 are not compared.) For example, two identical samples should have the same barcode. With increasing time, the fluctuations should randomize the fCpG methylation, and the probability of matching should be about 0.5, or the same as flipping a coin.

We compared FMCs between the top and bottoms of colon crypts (Fig. S2C). Crypt stem cells are located in the bottoms of crypts and cell differentiation occurs at crypt bottoms. Differentiated cells migrate upwards and the tops of crypts are differentiated cells that are lost within a week. As expected for a relatively short interval of a week, there was minimal switching between 0 to 1 or 1 to 0 between the top and bottom of the same crypt (Fig. S2D). This data also indicates the fluctuations in methylation pattern are not associated with cell differentiation.

By contrast, essentially a random distribution was found when FMCs were compared between adult crypts from different individuals or within an individual, with an average identity of barcodes of about 0.5 (Fig. S2E). The youngest individual was 17 years old, providing many years for methylation fluctuations to randomize between crypts that last share a common ancestor before birth. Consistent with a requirement for time for randomization, there was a trend for increasingly more random barcodes with age, with younger individuals tending to have more similar fCpG barcodes (Fig. S2F).

In summary, although we are unable to directly observe changes in the fCpG loci methylation, these observations are consistent with fluctuations in methylation at CpG sites with change occurring over many years. The fCpG sites do not switch during cell differentiation occurring over a week, and are rarely found in promoter regions or in highly expressed genes. Consistent with stochastic fluctuations that occur independently in different cells, FMC barcodes are essentially randomized between adult crypts from different individuals, and become increasingly different with aging within an individual. These findings are more consistent with dynamic methylation fluctuations that randomly change between 0, 0.5 and 1.0, rather than active regulatory oscillations or static random states. The FMCs distribution reflect the population structure of their cells. In stable polyclonal populations, the fCpG sites are asynchronous between cells and average methylation will be around 0.5, with narrow distributions as seen in normal whole blood. However, fCpG sites become synchronized in clonal populations and exhibit “W” shaped distributions, as seen in normal human crypts, endometrial glands and clonal hematopoiesis.

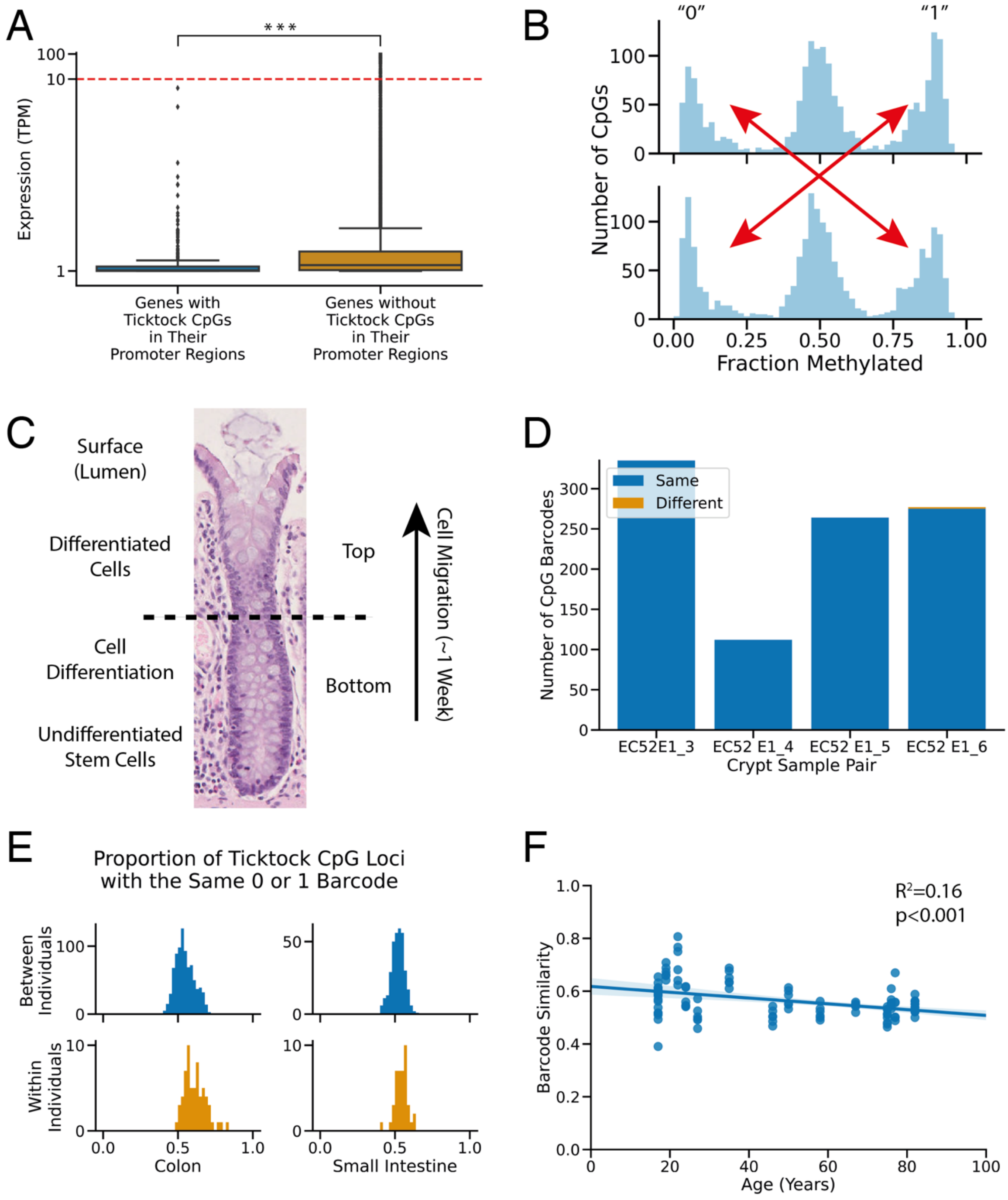

Supplementary Figure 2: Additional evidence supporting the existence of fluctuating CpG loci

A: RNA expression is on average 3 times lower ($p = 3.0 \times 10^{-28}$ two-sided Welch's t-test performed upon the log-transformed data) for genes with fCpG sites in their promoter regions compared to genes without promotor fCpG loci in the colon (RNAseq single cell data from GSE132257 - center line, median; box limits, upper and lower quartiles; whiskers, 1.5 IQR). **B:** FMC methylation reversibly fluctuates between 0, 50 and 100% methylated. Two samples can be compared by counting the number of fCpG loci that switch from 0 to 1 or vice versa. **C:** Colon crypts have small numbers of undifferentiated stem cells at their bases/bottoms. Cell differentiation occurs near crypt bottoms and fully differentiated cells migrate upwards and are lost within a week or two. **D:** Comparisons

between four top and bottom halves of the same crypts reveals FMC barcode methylation (0 or 1) is nearly always identical (986/988 barcode comparisons). **E:** Comparisons of colon and SI crypt FMC barcode methylation between and within individuals. The probability of another crypt having the same 0 or 1 barcode is approximately 0.5, or essentially random. **F:** Performing pairwise comparisons of the proportion of fCpG loci with the same 0 or 1 barcode, there was a slight trend towards increasingly random barcodes with age. Confidence band was calculated via bootstrapping and represents 95% confidence intervals.

Derivation of model describing methylation within the stem cell niche

Consider a single fCpG locus within a fixed population of S stem cells. Within each stem cell, the locus is assumed to be diploid, so each stem cell contains 2 alleles at this locus. In this way, there are 3 possible "states" for a given stem cell, (i) neither allele methylated, (ii) both alleles methylated, (iii) or one allele methylated whilst the other is unmethylated. We are interested in the population methylation level, so we assume that the population is well-mixed, which allows us to characterize the system using just 2 state variables: k – the number of stem cells containing a single methylated allele, and m – the number of stem cells containing 2 methylated alleles. The number of stem cells containing 0 methylated alleles is then given by $S - m - k$.

The states are constrained such that $0 \leq k, m \leq S$ and $k + m \leq S$, which allows us to calculate the total number of possible states by considering all possible combinations of k and m. If we first consider the case when $m = 0$, then k can take any value between 0 and S giving a total of $S + 1$ states. If we next consider the $m = 1$ case, then k can take any value between 0 and $S - 1$, a total of S states. We can continue in this fashion for each of the $S + 1$ possible states for m, such that the total number of states is

$$\sum_{m=0}^{S} S + 1 - m = \frac{1}{2}(S + 1)(S + 2) \tag{1}$$

We assume that there are three possible processes that can change the population methylation level $(k, m) \rightarrow (k', m')$:

(1) an unmethylated allele spontaneously becoming methylated (which, for a single unmethylated CpG locus, occurs at a rate μ per allele per stem cell per year)
(2) a methylated allele spontaneously becoming unmethylated (which occurs at a rate γ per allele per stem cell per year)
(3) one stem cell replacing one of the other $S - 1$ stem cells (which occurs at a rate λ per stem cell per year).

To formulate a system of differential equations that characterize the rates at which the population methylation changes, we first consider the probability the system in state (k, m) at time t transitions to state (k', m') within the time $t + \delta t$ (where we assume δt is small enough that the probability of a "double-jump" is negligible).

If we are in state (k, m), then the probability that one of the k heterozygous methylated stem cells becomes unmethylated (via process (2)) in a time period δt is:

$$\mathrm{P}\big((k, m) \rightarrow (k - 1, m)\big) = k\gamma\delta t \tag{2}$$

And the probability that the one of the m homozygous methylated stem cells (representing 2m methylated alleles) undergoes process (2) is:

$$\mathrm{P}\big((k,m) \rightarrow (k+1, m-1)\big) = 2m\gamma\delta t \tag{3}$$

Similarly, considering methylation (process (1)), there are a total of $2S - k - 2m$ unmethylated alleles where the process could occur. The probability that one of the homozygous S-k-m unmethylated stem cells becomes heterozygous is:

$$\mathrm{P}\big((k,m) \rightarrow (k+1, m)\big) = 2(S-k-m)\mu\delta t \tag{4}$$

And the probability that one of the heterozygous methylated stem cells becomes homozygous methylated is:

$$\mathrm{P}\big((k,m) \rightarrow (k-1, m+1)\big) = k\mu\delta t \tag{5}$$

Let us now consider the replacement process. In a time period δt the probability that a replacement occurs is $S\lambda\delta t$. There are $S(S-1)$ possible replacements: S possible cells that can expand, which must replace any of the $S-1$ other cells. To go from state (k,m) to a different state (k',m'), we require the expanding stem cell to replace a cell with a different methylation status. Therefore, the probability of the transition $(k,m) \rightarrow (k',m')$ is equal to the probability that any of the cells replaces another, $S\lambda\delta t$, multiplied by the number of ways that particular transition could occur, and normalized by the total possible number of transitions.

To give a concrete example, consider the stem the cell niche illustrated in Figure 1C, which contains 5 stem cells and is initially in the state $(k=3, m=1)$. There are a total of $5*4 = 20$ possible replacements. Clearly, if one of the heterozygous stem cells replaces another of the heterozygous stem cells, the population methylation level will not change. To jump to the state $(k=3, m=2)$ as illustrated in Figure 1C, only one replacement (the homozygous methylated stem cell replacing the homozygous unmethylated stem cell) allows the specified jump, hence the probability of the jump $(3,1) \rightarrow (3,2)$ in the time δt is $\frac{1}{5*4} 5\lambda\delta t = \frac{1}{4}\lambda\delta t$. To generalise this, the fraction of possible transitions that give rise to the particular jump $(k,m) \rightarrow (k',m')$ is equal to the multiplicity of the expanding cell multiplied by the multiplicity of the replaced cell, divided by $S(S-1)$.

Applying the same logic, we can derive the probability of all six possible state transitions via replacement:

$$\mathrm{P}\big((k,m) \rightarrow (k, m+1)\big) = \frac{m(S-m-k)\lambda\delta t}{S-1} \tag{6}$$

$$\mathrm{P}\big((k,m) \rightarrow (k+1, m)\big) = \frac{k(S-m-k)\lambda\delta t}{S-1} \tag{7}$$

$$\mathrm{P}\big((k,m) \rightarrow (k-1, m+1)\big) = \frac{km\lambda\delta t}{S-1} \tag{8}$$

$$\mathrm{P}\big((k,m) \rightarrow (k+1, m-1)\big) = \frac{km\lambda\delta t}{S-1} \tag{9}$$

$$\mathrm{P}\big((k,m) \to (k,m-1)\big) = \frac{m(S-m-k)\lambda\delta t}{S-1} \tag{10}$$

$$\mathrm{P}\big((k,m) \to (k-1,m)\big) = \frac{k(S-m-k)\lambda\delta t}{S-1} \tag{11}$$

The methylation switching and replacement processes that we have considered separately above are independent, allowing us to simply add the probabilities together (again, assuming that δt is small enough that the probability of two processes occurring in δt is negligible) to find the total probability that a given transition would occur:

$$\mathrm{P}\big((k,m) \to (k,m+1)\big) = \frac{m(S-m-k)\lambda\delta t}{S-1} \tag{12}$$

$$\mathrm{P}\big((k,m) \to (k+1,m)\big) = \frac{k(S-m-k)\lambda\delta t}{S-1} + 2(S-m-k)\mu\delta t \tag{13}$$

$$\mathrm{P}\big((k,m) \to (k-1,m+1)\big) = \frac{km\lambda\delta t}{S-1} + k\mu\delta t \tag{14}$$

$$\mathrm{P}\big((k,m) \to (k+1,m-1)\big) = \frac{km\lambda\delta t}{S-1} + 2m\gamma\delta t \tag{15}$$

$$\mathrm{P}\big((k,m) \to (k,m-1)\big) = \frac{m(S-m-k)\lambda\delta t}{S-1} \tag{16}$$

$$\mathrm{P}\big((k,m) \to (k-1,m)\big) = \frac{k(S-m-k)\lambda\delta t}{S-1} + k\gamma\delta t \tag{17}$$

We have considered above the transitions leading “out” of the state (k,m) into adjacent states (k',m'). However, we can also consider the jumps “into” the state (k,m) from the adjacent states (k',m'):

$$\mathrm{P}\big((k,m-1) \to (k,m)\big) = \frac{(m-1)(S-(m-1)-k)\lambda\delta t}{S-1} \tag{18}$$

$$\mathrm{P}\big((k-1,m) \to (k,m)\big) = \frac{(k-1)\big(S-m-(k-1)\big)\lambda\delta t}{S-1} + 2\big(S-m-(k-1)\big)\mu\delta t \tag{19}$$

$$\mathrm{P}\big((k+1,m-1) \to (k,m)\big) = \frac{(k+1)(m-1)\lambda\delta t}{S-1} + (k+1)\mu\delta t \tag{20}$$

$$\mathrm{P}\big((k-1,m+1) \to (k,m)\big) = \frac{(k-1)(m+1)\lambda\delta t}{S-1} + 2(m+1)\gamma\delta t \tag{21}$$

$$\mathrm{P}\big((k,m+1) \to (k,m)\big) = \frac{m(S-(m+1)-k)\lambda\delta t}{S-1} \tag{22}$$

$$\mathrm{P}\big((k+1,m) \to (k,m)\big) = \frac{(k+1)\big(S-m-(k+1)\big)\lambda\delta t}{S-1} + (k+1)\gamma\delta t \tag{23}$$

So far, we have considered the probability that the system changes from state (k,m) to state (k',m') within time δt. However, we primarily want to know the probability of the system being in state (k,m) at time t, $\mathrm{P}(k,m;t)$, and how this changes over time. For the system to be in state (k,m) at time $t+\delta t$, either (i) the system must have been in state (k,m) at time t and has not transitioned out of the state (which is equal to 1 minus the probability of transitioning to an adjacent state, defined by equations 12-17), (ii) or the system was in a

different (adjacent) state (k', m') at time t and has transitioned into the state (k, m) in time δt (defined by equations 18-23):

$$\begin{aligned}\mathrm{P}(k, m; t + \delta t) = \mathrm{P}(k, m; t)\left(1 - \sum_{k', m'} \mathrm{P}\big((k, m) \to (k', m')\big)\right) \\ + \sum_{k', m'} \mathrm{P}(k', m'; t)\mathrm{P}\big((k', m') \to (k, m)\big)\end{aligned} \tag{24}$$

We can rearrange equation 24, factoring out the common factor of δt in the $\mathrm{P}\big((k', m') \to (k, m)\big)$ terms and take the limit $\delta t \to 0$:

$$\begin{aligned}\frac{d\mathrm{P}(k, m; t)}{dt} &= \lim_{\delta t \to 0}\left(\frac{\mathrm{P}(k, m; t + \delta t) - \mathrm{P}(k, m; t)}{\delta t}\right) \\ &= \sum_{k', m'} \mathrm{P}(k', m'; t)\frac{\mathrm{P}\big((k', m') \to (k, m)\big)}{\delta t} \\ &\quad - \mathrm{P}(k, m; t)\ \frac{\mathrm{P}\big((k, m) \to (k', m')\big)}{\delta t}\end{aligned} \tag{25}$$

The sum over equation 12-17 in the final term evaluates as:

$$\begin{aligned}\sum_{k', m'} \frac{\mathrm{P}\big((k, m) \to (k', m')\big)}{\delta t} = \big(k(S - k) + m(S - k - m)\big)\frac{2\lambda}{S - 1} \\ + \big(2S - (k + 2m)\big)\mu + (k + 2m)\gamma\end{aligned} \tag{26}$$

Due to the constraints on k and m, we consider the differential equations for $(k = 0, m = 0)$, $(k = S, m = 0)$ and $(k = 0, m = S)$ separately. Combining equations 25, 26 and 18-23, we derive the following set of differential equations:

$$\frac{d\mathrm{P}(0,0|\lambda, \mu, \gamma; t)}{dt} = (\lambda + \gamma)\mathrm{P}(1,0|\lambda, \mu, \gamma; t) + \lambda\mathrm{P}(0,1|\lambda, \mu, \gamma; t) - S\mu\mathrm{P}(0,0|\lambda, \mu, \gamma; t) \tag{27}$$

$$\begin{aligned}\frac{d\mathrm{P}(S, 0|\lambda, \mu, \gamma; t)}{dt} &= (\lambda + 2\mu)\mathrm{P}(S - 1,0|\lambda, \mu, \gamma; t) \\ &\quad + (\lambda + 2\gamma)\mathrm{P}(S - 1,1|\lambda, \mu, \gamma; t) \\ &\quad - S(\mu + \gamma)\mathrm{P}(S, 0|\lambda, \mu, \gamma; t)\end{aligned} \tag{28}$$

$$\begin{aligned}\frac{d\mathrm{P}(0, S|\lambda, \mu, \gamma; t)}{dt} &= (\lambda + \mu)\mathrm{P}(1, S - 1|\lambda, \mu, \gamma; t) \\ &\quad + \lambda\mathrm{P}(0, S - 1|\lambda, \mu, \gamma; t) \\ &\quad - S\gamma\mathrm{P}(0, S|\lambda, \mu, \gamma; t)\end{aligned} \tag{29}$$

Otherwise:

$$
\begin{aligned}
&\frac{d\mathrm{P}(k,m|\lambda,\mu,\gamma;t)}{dt} \\
&= \big(S-m-(k-1)\big)\left((k-1)\frac{\lambda}{S-1}+2\mu\right)\mathrm{P}(k-1,m|\lambda,\mu,\gamma;t) \\
&+(m-1)(S-(m-1)-k)\frac{\lambda}{S-1}\mathrm{P}(k,m-1|\lambda,\mu,\gamma;t) \\
&+(k+1)\left((m-1)\frac{\lambda}{S-1}+\mu\right)\mathrm{P}(k+1,m-1|\lambda,\mu,\gamma;t) \\
&+(k+1)\left(\big(S-m-(k+1)\big)\frac{\lambda}{S-1}+\gamma\right)\mathrm{P}(k+1,m|\lambda,\mu,\gamma;t) \\
&+(m+1)(S-(m+1)-k)\frac{\lambda}{S-1}\mathrm{P}(k,m+1|\lambda,\mu,\gamma;t) \\
&+(m+1)\left((k-1)\frac{\lambda}{S-1}+2\gamma\right)\mathrm{P}(k-1,m+1|\lambda,\mu,\gamma;t) \\
&-(2\big(k(S-k)+m(S-k-m)\big)\frac{\lambda}{S-1}+\big(2S-(k+2m)\big)\mu \\
&\quad +(k+2m)\gamma)\mathrm{P}(k,m|\lambda,\mu,\gamma;t)
\end{aligned}
\tag{30}
$$

This master equation determines how the methylation statues of a single CpG locus within the stem cell niche evolves over time. The replacement, methylation and demethylation rate are assumed to be constant, hence this process is Markovian and we are able to solve this using standard matrix exponentiation.

Bayesian analysis of the effect of tissue location and disease state on stem cell dynamics

The Bayesian pipeline described in the main body of the text allowed the posterior distribution of the parameters defining the stem cell dynamics (namely, the effective number of stem cells, S, and the replacement rate per stem cell, λ) of each individual crypt to be inferred. To interrogate the effect of age, sex, tissue location (colon, small intestine and endometrium) and the disease state of colonic crypts (AFAP/FAP) on stem cell dynamics, we take the posterior mean of S and λ as representative of the inferred distribution for each crypt.

As a matter of notation, let there be K patients subscripted with $k = [1\,..\,K]$ and N crypts subscripted with $i = [1\,..\,N]$. The age of the kth patient is t_k, which we normalise to be between 0 and 1 by dividing each patient's age by the maximum age in the patient cohort. Similarly, the sex the kth patient is encoded as a dummy variable, which equals 0 for female patients and 1 for male patients. The location/disease state of each crypt is encoded with the dummy variables $x_{i,j}$ for $j \in \{Colon, Small\ Intestine, FAP,\ AFAP, Endometrium\}$.

We fit the parameters determining stem cell dynamics $y = \{S, \lambda\}$ using a generalised linear model with a gamma-distributed dependent variable (this accounts for the fact S and λ are strictly positive). Let $y_{i,k}$ be the dependent variable with expectation $\hat{y}_{i,k}$, then we employ the natural log as a link function $g(\hat{y}_{i,k}) = \ln(\hat{y}_{i,k})$. $y_{i,k}$ is then gamma distributed with mean $\hat{y}_{i,k}$ and a tissue/disease-specific standard deviation ϕ_j.

We use the parameterization of the gamma distribution in terms of its shape (ψ) and rate (ω):

$$\text{Gamma}(y|\psi,\omega) = \frac{\omega^{\psi}}{\Gamma(\psi)} y^{\psi-1} e^{-\omega y}$$

The mean of this distribution is $\frac{\psi}{\omega}$ and the variance is $\frac{\psi}{\omega^2}$. Hence, to parameterize the gamma distribution in terms of its mean ($\hat{y}$) and standard deviation (ϕ), we apply the transformation $\psi = \frac{\hat{y}^2}{\phi^2}$, $\omega = \frac{\hat{y}}{\phi^2}$.

Our dataset contains multiple samples from the same patient, so we assume the offset in the linear predictor is drawn for each patient from a hierarchical normal distribution with mean ν and variation σ (hence accounting for random inter-patient variability, not attributable to the factors we are explicitly modelling). Similarly, to maximize the information that can be drawn from the data, we allowed the tissue/disease-specific intrapatient standard deviation, ϕ_j, to be drawn from a lognormal distribution, with a population mean ρ and standard deviation ζ.

Priors:

$$a_k \sim \text{normal}(\nu,\ \sigma)$$

$$\ln(\phi_j) \sim \text{normal}(\rho, \zeta)$$

Model:

$$\ln(\hat{y}_{i,k}) = a_k + b_j {x_i}^j + \ c t_k + d s_k$$

$$y_{i,k} \sim \text{gamma}\left(\frac{{\hat{y}_{i,k}}^2}{{\phi_j}^2}, \frac{\hat{y}_{i,k}}{{\phi_j}^2}\right)$$

The hierarchical Bayesian model was fit to the data using pystan, a python implementation of Stan[5], a probabilistic programming language that allows for rapid MCMC sampling.

Because a log-link function was used to ensure the positivity of $\hat{y}_{i,k}$, the coefficients of the regression, b_j, encode the difference between each tissue-type or disease-state, and colon on the log scale. We take the exponential transform of each of these regression coefficients to derive the posterior for the relative stem cell number and replacement rate of each tissue-type and disease-state relative to colon. We take a hypothesis testing by parameter inference approach, where the effect of a particular tissue/disease on the dependent variable is termed significant when the 95% equal-tailed credible interval does not overlap 0. The hierarchical Bayesian model that we have developed naturally penalizes increasing numbers of parameters, hence there is no need to apply a multiple test correction [6].

Investigating the well-mixed assumption

One of the major assumptions taken in the development of the mathematical model describing the FMC distribution was that the stem cells within the niche are well-mixed; that is, each stem cell can replace any other stem cell with equal probability. This assumption was made to minimize the mathematical and computational complexity of the model, as it allowed the state of the stem cell system to be fully characterized with just 2 state variables. However, previous work in mouse suggests that stem cells within the crypt are organized into a ring-like structure, where each stem cell can only replace the 2 cells directly neighboring it (Fig. S3A). It is worth noting that the replacement rate in mouse is on the order of months[7], whereas in human the replacement rate is on the order of years[8]. Hence the potential of stem cells to randomly swap position, as identified by Ritsma *et al.*[9], raises the possibility that within human crypts, the most accurate model of stem cell replacement is neither perfectly well-mixed nor perfectly organized into a ring.

To investigate the effect of this well-mixed assumption, a Gillespie simulation was developed to model the stem cell replacement process for the well-mixed and ring geometry (code accessible at https://github.com/CalumGabbutt/flipflop.git[10], see gillespie_crypt.py). Briefly, we generated a 3-dimensional binary array of size $[S, 2, n]$ to model the stem cell niche. The time until the next replacement event was drawn from a Poisson distribution by generating a random uniform number $r \sim \text{uniform}(0, 1)$ as follows:

$$\Delta t = \frac{1}{\lambda S} \log\left(\frac{1}{r}\right)$$

We accounted for the (de)methylation of the individual CpG loci by recognizing that each individual CpG locus on a particular DNA strand was effectively a 2-state system with forward rate μ and reverse rate γ. Given that a given CpG locus is methylated at time t, the probability that the CpG locus is still methylated at time $t + \Delta t$ is:

$$P(on|on) = \frac{\mu}{\mu + \gamma} + \left(1 - \frac{\mu}{\mu + \gamma}\right) e^{-(\mu+\gamma)\Delta t}$$

Whilst the probability that the same locus is not methylated is:

$$P(off|on) = 1 - P(on|on)$$

Similarly, the probability that a given CpG locus that is demethylated at time t is methylated at $t + \Delta t$ is:

$$P(on|off) = \frac{\mu}{\mu + \gamma}\left(1 - e^{-(\mu+\gamma)\Delta t}\right)$$

And the probability that that CpG locus is still demethylated at time $t + \Delta t$ is:

$$P(off|off) = 1 - P(on|off)$$

Hence, once the time until the next replacement had been drawn, we could update the methylation states of the individual CpG sites by drawing new methylation states with the aforementioned probabilities. The replacement could then be handled by choosing one cell to clonally expand and another to recede. Depending on the geometry of the crypt that the simulation was intended to model, this could be done by selecting one cell at random and then selecting one of that cell's neighbors with equal probability (ring model), or by selecting two cells at randomly without replacement (well-mixed model). To ensure that that our analysis was probing the effect of differing geometry, in each case 100 synthetic crypts were generated and the average methylation probability distribution determined.

For crypts containing $S = 5$ stem cells which replace each other at a rate $\lambda = 1.0$ replacements/stem cell/year. The average FMC distribution of the ring simulations is not significantly different from predictions of the well-mixed model (Fig. S3B, $p > 0.05$ Kolmogorov–Smirnov test).

The impact of the geometry with increasing numbers of stem cells on the inference performed upon the methylation distribution was also explored. The above simulations were replicated for $S = 10$ and $S = 15$ for both ring and well-mixed geometries, and the Bayesian inference framework was run upon each of the average methylation distributions. At low values of S, the inference model was able to accurately recover the known stem cell dynamics parameters; however, for higher values of S the inferred replacement rate of the

ring simulations was lower than the ground truth value (Fig. S3C). The majority of samples analyzed in this study had an inferred stem cell number lower than 10, hence, the impact of the well-mixed assumption was likely to be minimal.

This result intuitively aligns with our understanding of the mean fixation time (the average time for a mutation that occurs at $t = 0$ to sweep through the population and become clonal, conditioned upon survival), at low stem cell number the two geometries have similar fixation times; however, the fixation time for the ring model scales as $\sim S^2$, whereas the fixation time for the well-mixed model only scales $\sim S$ (Fig. S3D). In the case of a ring geometry with a large number of stem cells, the mean fixation time is larger than for the well-mixed case, and hence there are a greater number of subclonal fCpG loci, which the inference model accounts for by proposing a lower inferred replacement rate.

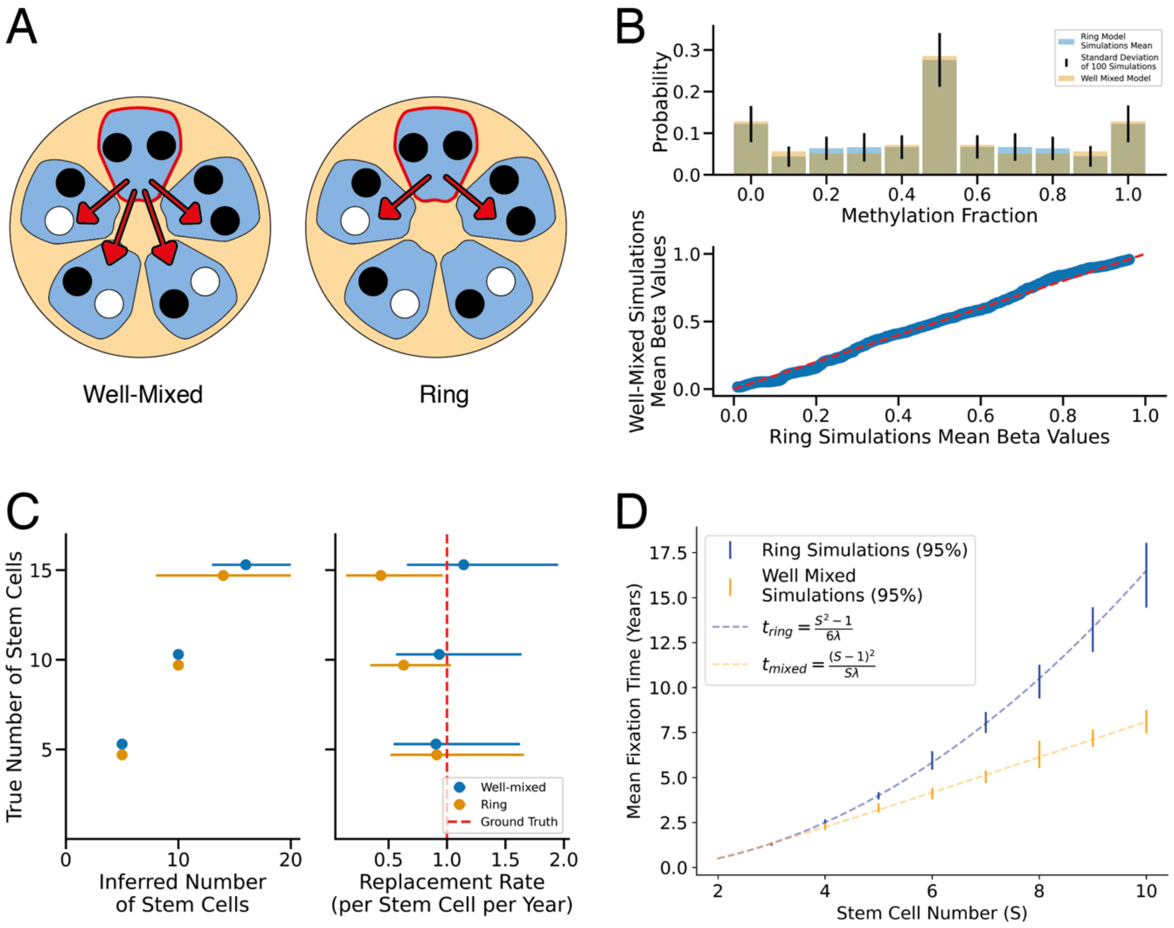

Supplementary Figure 3: Well-mixed vs. ring stem cell geometry

A: To test the effect of the stem cell geometry on the resulting FMC distribution and the inference process, a simulation of the crypt stem cell dynamics was developed with either a well-mixed (each cell can replace any other) or ring (each cell can only replace its neighbors) geometry. **B:** (top) A histogram displaying the discrete methylation fraction distribution (i.e. before sampling/technical noise has been added) for a set of 100 simulations of $S = 5$ stem cells arranged in a ring geometry, with the well-mixed model predictions overlaid. Error bars denote 1 standard deviation. (bottom) A Q-Q plot comparing the average methylation fraction distribution of the set of 100 ring simulations against that of 100 well-mixed distributions. The two distributions are not statistically different ($p > 0.05$ two-sided KS test). **C:** The 95% credible interval of the posterior for the

number of stem cells (left) and replacement rate (right) compared to the ground truth. **D:** A modified version of the simulation was designed to track the fixation time of a mutation introduced at $t = 0$ to fix within the population. The resulting mean fixation time (10000 total simulations per stem cell number), conditioned on survival, is plotted against the number of stem cells, with the analytic predictions of each model overlaid ($t_{ring} = \frac{S^2-1}{6\lambda}$, $t_{mixed} = \frac{(S-1)^2}{S\lambda}$). Bars denote 95% confidence intervals.

Non-fluctuating CpG loci

In the development of our inference framework, one of the implicit assumptions was that every CpG loci that we had identified as an fCpG was actually behaving in a fluctuating manner. However, whilst every precaution was taken to filter out CpGs which were likely to be under active selection/regulation (see main text), there remained the possibility that a fraction of the identified CpGs do not truly fluctuate. CpG loci that do not behave in a clocklike manner will not track the clonal dynamics of the stem cells, and therefore will dilute the timing signal of the fCpGs.

To investigate the effect that this would have upon our inference framework, we first generated a synthetic crypt containing $S = 5$ stem cells, with a replacement rate $\lambda = 1.0$ replacements/ stem cell/year and a (de)methylation rate $\mu = \gamma = 0.05$ per allele/stem cell/year. The noise due to sampling was then simulated so that each of the resulting samples were identical with respect to the fCpG loci using the transforms specified in the main text ($\delta = 0.04, \epsilon = 0.92, \kappa = 200$). Then we replaced a fraction of the fCpGs, $\omega = \{0, 0.05, 0.1, \ldots, 0.9, 0.95, 1\}$ with CpGs that were randomly assigned a beta value of either 0 or 1 with equal probability (with noise added such that the non-fluctuating CpG loci had the same noise profile as the fCpG sites).

The inference framework pipeline was run upon each of the 11 resulting synthetic crypts (example posteriors for $\omega = 005, 0.25, 0.5$ are presented in Fig. S4A). The effect of non-fluctuating CpG loci was to effectively reassign clonal heterozygous and subclonal CpGs to clonal homozygous states. For $\omega \leq 0.5$, there are still sufficient subclonal mutations to accurately infer the number of stem cells (Fig. S4B). Initially, the effect of the relabeling on the inferred parameters was to decrease both the (de)methylation rate and the replacement rate, effectively assuming that the methylation distribution has not relaxed as far from the initial conditions (Fig. S4C). However, once $\omega > 0.5$, the inference struggles to infer the number of stem cells, and the uncertainty over S is propagated into the posterior for λ due to the correlations in the posterior between λ and S.

This modelling applies equally well to mistakenly identifying fCpG loci that do not fluctuate and are static over time, and the possibility that a fraction of fCpG loci are actively regulated, dynamically setting the methylation status of all the stem cells in the crypt at that locus to be the same. Note that our analysis of fCpG loci located on chromosome X (Fig. S1D) and our finding that fCpG methylation status is preserved along the crypt (Fig. 2C) suggests the influence of cell-type specific methylation is unlikely to be major.

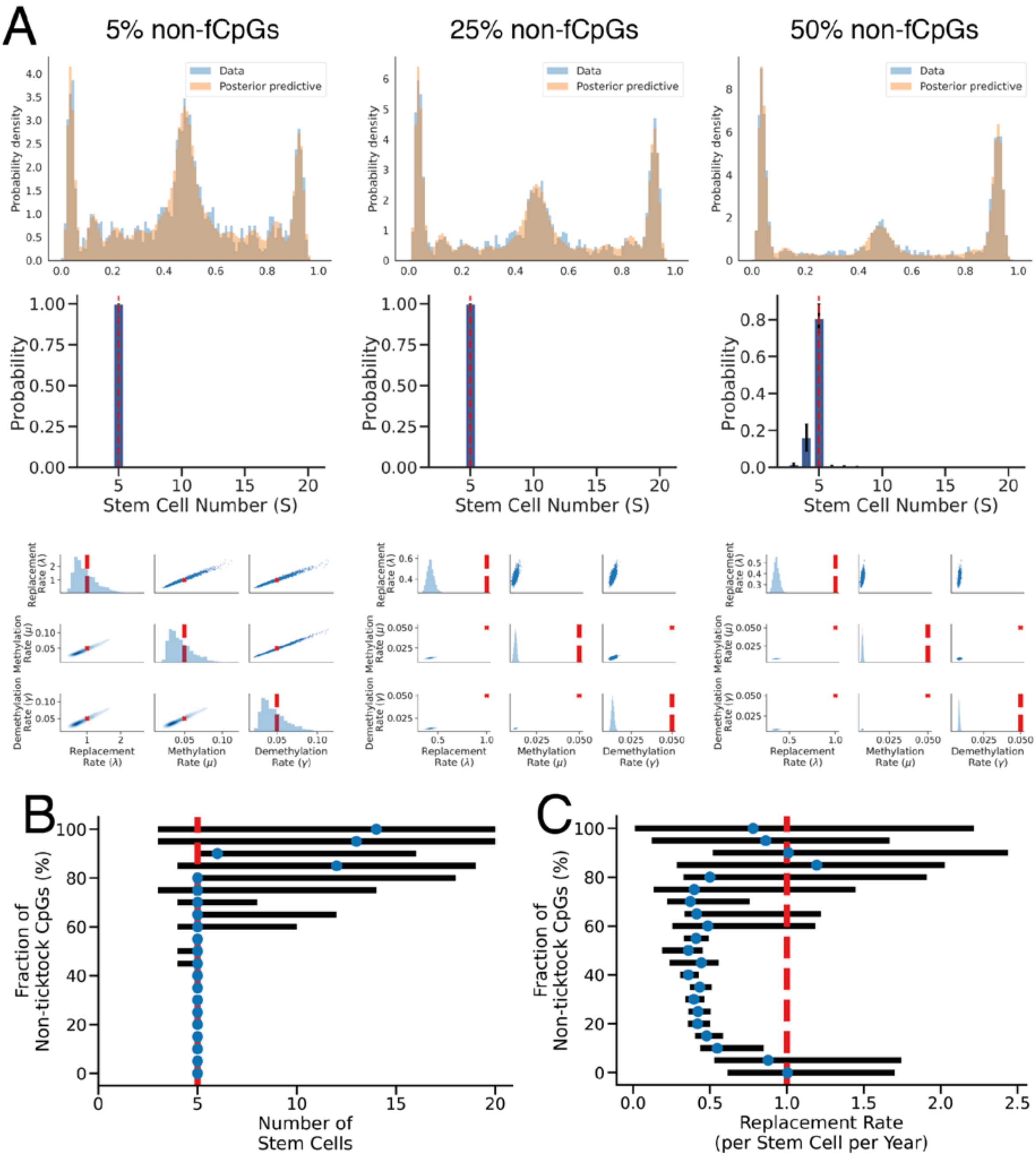

Supplementary Figure 4: Non-fluctuating CpG loci

To investigate the effect of a fraction of the CpG sites that we have identified as FMCs in fact being under active regulation, we generated synthetic crypts with an increasing fraction of non-fluctuating CpG loci and ran the inference framework upon them.

A: Example posterior predictive distributions and posterior distributions for 5, 25 and 50% of non-fCpGs. (middle) Error bars were calculated from the estimated error (1 standard deviation) on the log-evidence. **B-C:** 95% credible intervals for the inferred replacement rate and stem cell number respectively for an increasing fraction of non-fluctuating CpG sites.

Mean fixation time

For small populations of cells in a process of neutral competition, one cell stochastically clonally expanding until it dominates the niche is an inevitability; however, the time-scale over which this process occurs varies depending on both the replacement rate per stem cell and the number of stem cells within the niche. The time it takes for a mutation appearing within a single cell to undergo monoclonal convergence, conditioned upon that mutation not going extinct, follows a positive skewed distribution (Fig. S5A); however, this distribution is often summarized using a single statistic – the mean fixation time.

For a ring geometry, where stem cells can only replace their neighbors, the mean fixation time scales $\sim\frac{S^2}{\lambda}$, whereas for a well-mixed geometry the mean fixation time scales $\sim\frac{S}{\lambda}$ (Fig. S3D). Our inference framework relies upon a well-mixed geometry, and therefore for reasons of self-consistency we shall assume the formula for the mean fixation time is $t_{mixed} = \frac{(S-1)^2}{\lambda S}$.

For each crypt sample, for every point in the posterior we calculated the mean fixation time to generate a corresponding posterior for the mean fixation time. We followed the same hierarchical Bayesian generalized linear model as in the main text. The only significant factor was the age of the patient (Fig. S5B), suggesting that the rate at which a stem cell colonizes a crypt slows down over the course of a patient's lifetime. We present the mean fixation time for all of the intestinal crypts in Fig. S5C.

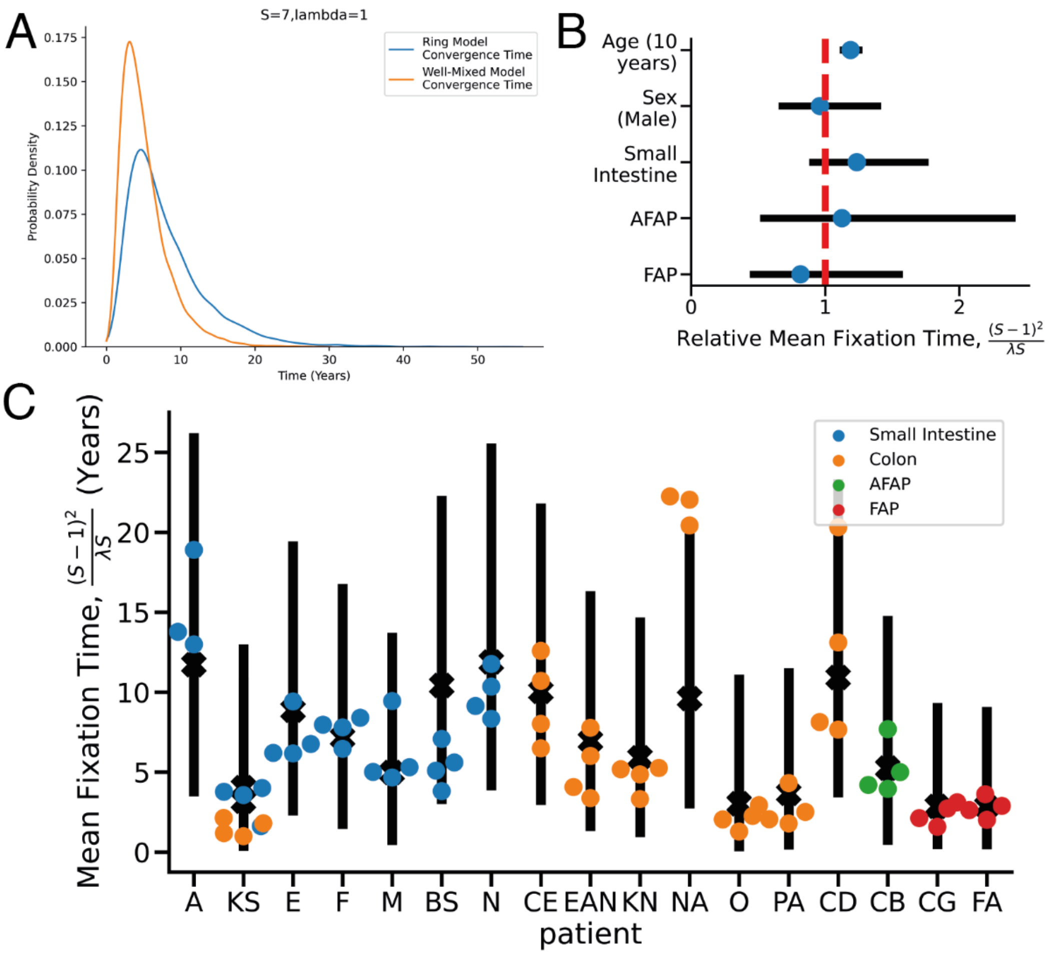

Supplementary Figure 5: Mean fixation time

A: The time for a mutation introduced at $t = 0$ to "fix" within the population, conditioned upon that mutation not going extinct, was simulated using the same simulations outlined in "Supplementary Materials - Investigating the Well-Mixed Assumption". The fixation time follows a skewed distribution, with different shapes depending upon the geometry of the stem cells population. **B:** posterior distributions (95% credible intervals) for the effect of patient age (per decade), sex (with female encoded as reference), tissue type and disease state on the relative mean fixation time compared to normal colon. **C:** individual crypt and posterior mean per patient for the mean fixation time, with the 95% credible range of the generalized linear model (GLM) expectation, accounting for age, sex, tissue, disease state and intra- and inter-patient heterogeneity.

Linkage between CpG Loci

The mathematical model describing how the distribution of methylation patterns evolves over time presented in the main text treats each fCpG locus independently; however, the replacement process will couple the methylation status of individual CpG loci (because a cell contains a set of genetically "linked" CpGs). Unlike traditional unidirectional lineage tracing markers (e.g. SNVs), the relabeling of individual CpG loci will cause these correlations to naturally erode over time. To investigate the effect that these correlations between individual CpG sites will have upon the FMC distribution, we employed the well-mixed Gillespie simulations described above (Investigating the Well-Mixed Assumption) to generate 100 synthetic crypts ($S = 5, \lambda = 1.0, \mu = \gamma = 0.05$) which will naturally include intra- CpG correlations. The resulting probability mass functions (PMF) were compared to 100 draws from the analytic model. The mean PMF of the synthetic crypts exactly matched that of the analytic model (Fig. S6), but the crypt simulations exhibited a wider degree of variability than would be expected from the analytic model alone.

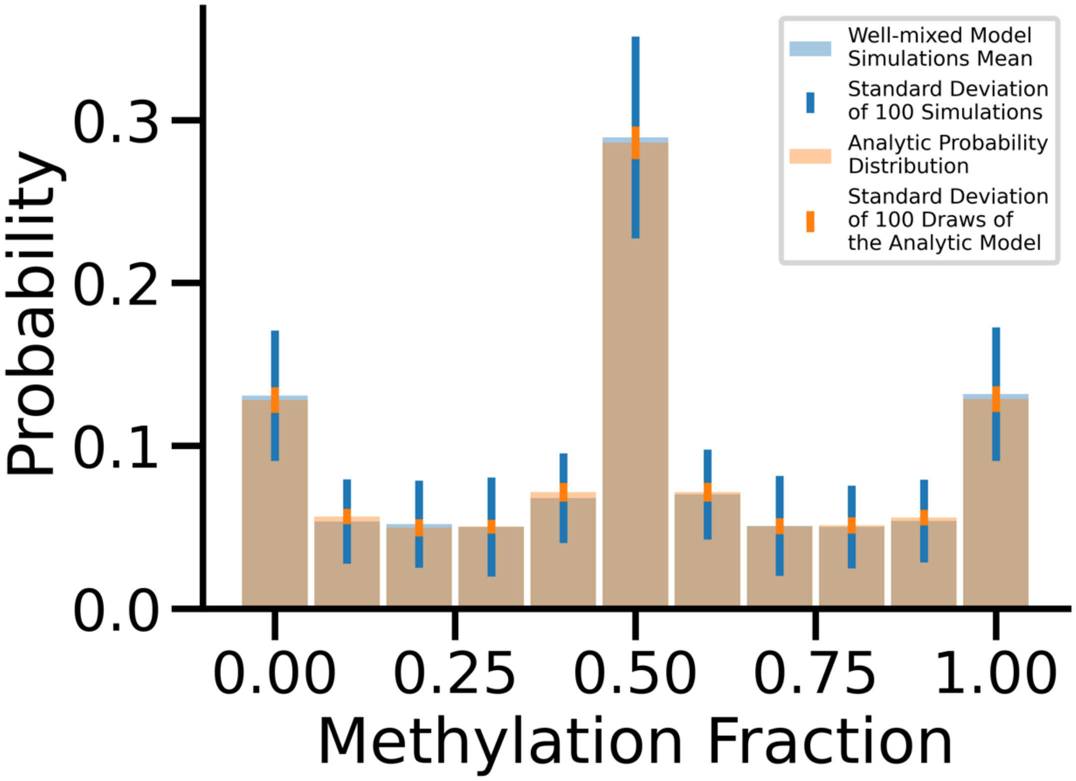

Supplementary Figure 6: Correlations Between Different fCpG Loci

The analytic model derived in the text assumes that individual CpG loci behave independently; however, the replacement process will correlate the fates of CpG loci located on separate chromosomes. To investigate this, 100 simulations of well-mixed crypt were performed, and the mean and the standard deviation of the methylation recorded for each possible methylation state. Similarly, the analytic probability distribution was sampled 100 times. Error bars denote 1 standard deviation. The effect of these correlations is that the mean methylation distribution of the simulated crypts exactly matches that of the analytic model, but that the simulated crypts had a higher degree of intra-crypt variability than the analytic model predicts.

Identifiability of rate parameters

The posterior of the inference presented in the main text exhibits a degree of collinearity between μ, γ and λ (Fig. 3A, 4A and 5B). To demonstrate that the system is sensitive to the absolute values of the rate parameters, rather than just their relative values, the methylation distribution was generated for an identical crypt to the “just right” crypt generated in Fig. 3, but with all the rate parameters 10 times smaller (i.e. $\lambda = 0.1$ replacements/stem cell/year, $\mu = \gamma = 0.005$ per CpG locus/year). The resulting methylation was markedly different (Fig. S7A), lacking the clonal heterozygous peak at 0.5 as the system has not yet decayed far from the initial conditions.

The intuition behind this result, and the reason why the absolute rate parameters are obtainable, is because of the slow speed at which the model converges to the steady state. The matrix exponentiation step discussed above can be re-written in terms of the eigenvalues (u_i) and eigenvectors ($\vec{v}_i$) of the transition matrix.

$$\vec{P}(t) = e^{t\boldsymbol{T}}\vec{P}(t=0) = \sum c_i e^{u_i t}\vec{v}_i$$

The system decays from the initial state according to the magnitude of the non-zero eigenvalues (which are all negative) towards the steady state (the eigenvector corresponding to the eigenvalue with 0 magnitude). Therefore, the smallest magnitude non-zero eigenvalue determines how rapidly the system decays to the steady state.

The eigenvalues can be calculated numerically for a given set of parameters $\{\lambda, \mu, \gamma, S\}$. If we use the same set of parameters as in the "just right" simulated data (Fig. 3, $\{\lambda = 1.0, \mu = 0.05, \gamma = 0.05, S = 5\}$), then the smallest magnitude non-zero eigenvalue has a value of -0.1 /year. After 30 years, the slowest eigenvalue will have decayed to $e^{-3} \approx 0.05$ of its initial value – certainly low, but sufficient for the inference model to accurately infer the replacement rates in real units, as exhibited in Fig. 3. When we scale each of the rate parameters to be 10 times smaller, as presented in Fig. S7A, then although the ratio of the replacement:methylation:demethylation rates are unchanged, the system has decayed much less towards the steady state, which the inference framework is able to detect and quantify. This reinforces the importance of selecting fCpG loci with a (de)methylation rate that is "just right". If sites (de)methylate too quickly, the system is indistinguishable from the steady state distribution and the information on the absolute values of the rate parameters is lost (as in the "too fast" synthetic crypt in Fig. 3). If sites (de)methylate too slowly, the methylation state of the system does not evolve sufficiently over the observed time period.

We note that in the real patient data, the mean inferred methylation rate is 0.027 across the sample cohort, approximately half that of the assumed value in the simulated data, and so the decay to steady state in patient is slower than presented in the simulations in Fig 3.

To ensure that the parameters were separately identifiable, the inference on the "just right" simulated data (Fig. 3) was repeated with a wider prior on the (de)methylation rate ($\mu, \gamma \sim \mathrm{halfnormal}(0.5)$). The 95% credible interval still contained the ground truth parameters values and the posterior predictive distribution well-matched the data (Fig. S7B-D), hence the parameters were separately identifiable.

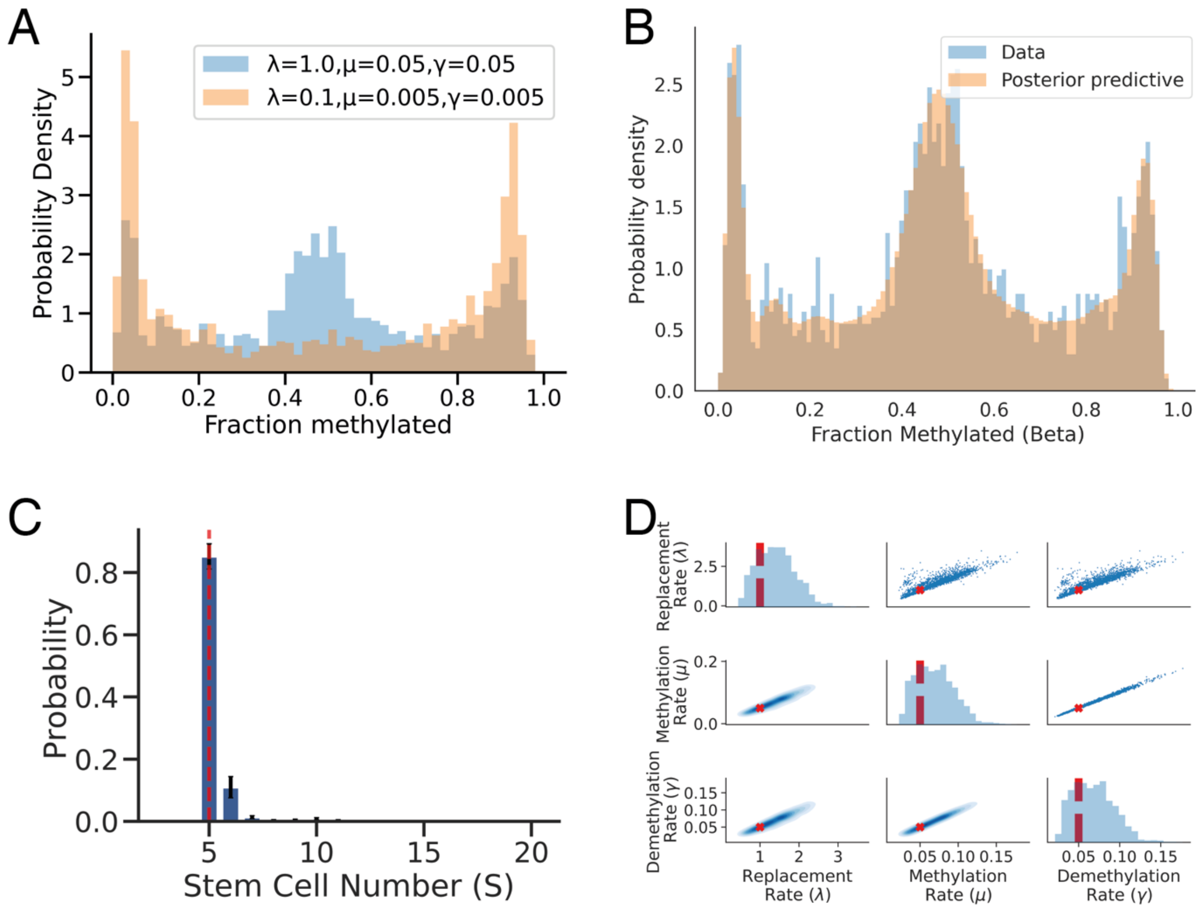

Supplementary Figure 7: Identifiability of parameters

A: Example methylation distributions for identical crypts, except with the rate parameters of one crypt 10 times smaller than the other. The resulting methylation distributions are strikingly different. **B-C** To demonstrate that the parameters are separately identifiable, the inference upon the "just right" synthetic crypts (Fig. 3) was repeated with 10 times wider priors upon the (de)methylation rates. The resulting posterior is still able to accurately recover the ground truth values. Error bars were calculated from the estimated error (1 standard deviation) on the log-evidence.

Whole blood simulations

Whole blood was simulated in Java using the HAL framework[11] as a non-spatial agent-based model using 27,634 fCpG sites as measured in the experimental data. Parameters (Supplemental Table 1) for normal hematopoiesis are numbers of hematopoietic stem cells (N, HSCs), number of possible division events (T), CpG error rates (S, methylation and demethylation) for the fCpG sites, and HSC replacement dynamics (λ). To model clonal expansion, a single cell was selected to grow upon induction, and added parameters are its expansion rate (E) and its final blood frequency of the clonal expansion (ω). These clonal expansions resulted in the overall population size to grow until the appropriate final blood frequency was reached. The output of the simulations provided the beta values at the fCpG sites and the overall distribution variance over time.

The number of HSCs was set at a lower value of 1000 initiating cells. This was much lower than the 30,000 based on the large number of HSC inferred by DNA sequencing studies[12,13]; however, the results shown here are invariant to more than 100 initiating cells. CpG error

rates varied between CpG sites and were assigned based on the distribution averages of the 656 normal individuals from GSE40279[14]. We found that some of the whole blood fCpGs did not appear to have equal methylation and demethylation error rate because their averages tended to always be above or below 50% in multiple individuals. Hence, to better model and match the data, we used a look-up distribution table in the simulations in order to initialize a cell's fCpG parameters, with lower and unequal error rates at CpG sites with average methylation typically found near 0.4 (demethylation > methylation) or near 0.6 (methylation > demethylation) to maintain the variance of the 27,634 fCpG sites around 0.1 during cell divisions. The error rates varied between 0.0001 to 0.001 changes per division, with the highest error rates and more equal methylation and demethylation rates at CpG sites near 50% methylation.

Cell survival was set at exact replacement (one cell produces one living offspring), and results did not vary much if random replacement was simulated. A proportion of cells underwent replacement at each timestep (Supplemental Table 1). For the neoplastic simulations in Fig 6D in the manuscript, the expansion rate (E) was varied to model either rapid expansion (visible or more than 5% leukemic cells within 1 year or 200 divisions) akin to acute leukemia, modest expansion (visible within 4 years or 12,000 divisions), or very slow expansion (visible within 6 years or 18,000 divisions). The extent of blood involvement was varied between 20% (black lines), 50% (blue lines) and 90% (red lines). These simulations indicated that how clonal expansions change whole blood fCpG variances depends both on how fast the expansion grows and to what extent it involves the blood. Rapid growth to high levels like acute leukemias results in high fCpG variances and characteristic W-shaped fCpG distributions. Slower growth to lower levels like chronic leukemias results in low fCpG variances and broader distributions that lack the W-shape. Interestingly, very indolent clonal expansions which may occur with CHIP[15] can result in small increases in fCpG variances, which may account for the age-related increase in fCpG variances seen in Fig 6A in the manuscript.

More sophisticated modelling with a better selection of whole blood fCpG sites could improve the extraction of ancestral information. For example, a selection of slower fCpG sites may improve the detection and analysis of indolent clonal expansions, where many of the faster fluctuations return to average ~50% methylation by the time the expansion reaches detectable blood levels.

The simulation framework can be obtained, along with sample simulation results, on GitHub through https://github.com/MathOnco/flipflopblood.git[16]. A GUI compatible with most operating systems is accompanied to allow for rapid evaluation of different parameters.

Parameter	Description	Values
N	Number of HSCs, initial population	100
T	Simulation time	2,500
S	CpG error rates	0.0001-0.001 per division
λ	Cell survival, exact replacement	0.6
E	Disease expansion rate	(0.1, 0.005, 0.00225)
ω	Final blood frequency	(0.2, 0.5, 0.9)

Supplementary Table 1: Parameters of whole blood simulations

Parameters used in simulations describing how the methylation distribution of well-mixed hematopoietic stem cells (HSCs) changes in response to the expansion of a single clonal population.

Supplementary References

1. Métivier, R. *et al.* Cyclical DNA methylation of a transcriptionally active promoter. *Nature* **452**, 45–50 (2008).
2. Kangaspeska, S. *et al.* Transient cyclical methylation of promoter DNA. *Nature* **452**, 112–115 (2008).
3. Rulands, S. *et al.* Genome-Scale Oscillations in DNA Methylation during Exit from Pluripotency. *Cell Syst.* **7**, 63-76.e12 (2018).
4. Parry, A., Rulands, S. & Reik, W. Active turnover of DNA methylation during cell fate decisions. *Nature Reviews Genetics* vol. 22 59–66 (2021).
5. Carpenter, B. *et al.* Stan: A Probabilistic Programming Language. *J. Stat. Software; Vol 1, Issue 1* (2017).
6. Gelman, A., Hill, J. & Yajima, M. Why We (Usually) Don't Have to Worry About Multiple Comparisons. *J. Res. Educ. Eff.* **5**, 189–211 (2012).
7. Kozar, S. *et al.* Continuous Clonal Labeling Reveals Small Numbers of Functional Stem Cells in Intestinal Crypts and Adenomas. *Cell Stem Cell* **13**, 626–633 (2013).
8. Nicholson, A. M. *et al.* Fixation and Spread of Somatic Mutations in Adult Human Colonic Epithelium. *Cell Stem Cell* **22**, 909-918.e8 (2018).
9. Ritsma, L. *et al.* Intestinal crypt homeostasis revealed at single stem cell level by in vivo live-imaging. *Nature* **507**, 362–365 (2014).
10. Gabbutt, C. *et al.* Cell lineage tracing with molecular clocks based on fluctuating DNA methylation - flipflop. *Zenodo* (2021) doi:10.5281/zenodo.5347259.
11. Bravo, R. R. *et al.* Hybrid Automata Library: A flexible platform for hybrid modeling with real-time visualization. *PLoS Comput. Biol.* **16**, e1007635 (2020).
12. Lee-Six, H. *et al.* Population dynamics of normal human blood inferred from somatic mutations. *Nature* **561**, 473–478 (2018).
13. Watson, C. J. *et al.* The evolutionary dynamics and fitness landscape of clonal hematopoiesis. *Science (80-.).* **367**, 1449–1454 (2020).
14. Hannum, G. *et al.* Genome-wide Methylation Profiles Reveal Quantitative Views of Human Aging Rates. *Mol. Cell* **49**, 359–367 (2013).
15. Jaiswal, S. & Ebert, B. L. Clonal hematopoiesis in human aging and disease. *Science (80-.).* **366**, eaan4673 (2019).
16. Schenck, R. *et al.* Cell lineage tracing with molecular clocks based on fluctuating DNA methylation – Flip flop blood model. *Zenodo* (2021) doi:10.5281/zenodo.5348301.
