# Supplementary material for: Fluctuating methylation clocks for cell lineage tracing at high temporal resolution in human tissues
Source: Nat Biotechnol. 2022 Jan 3;40(5):720–30. doi: 10.1038/s41587-021-01109-w (PMC9110299; doi:10.1038/s41587-021-01109-w)
Supplement: Supplementary file 2 — Reporting Summary. [file 41587_2021_1109_MOESM2_ESM.pdf]

nature research

Corresponding author(s): Alexander RA Anderson, Trevor A Graham, Darryl Shibata

Last updated by author(s): Aug 27, 2021

Reporting Summary

Nature Research wishes to improve the reproducibility of the work that we publish. This form provides structure for consistency and transparency in reporting. For further information on Nature Research policies, see our Editorial Policies and the Editorial Policy Checklist.

Statistics

For all statistical analyses, confirm that the following items are present in the figure legend, table legend, main text, or Methods section.

n/a	Confirmed	
☐	☒	The exact sample size (*n*) for each experimental group/condition, given as a discrete number and unit of measurement
☐	☒	A statement on whether measurements were taken from distinct samples or whether the same sample was measured repeatedly
☐	☒	The statistical test(s) used AND whether they are one- or two-sided<br>*Only common tests should be described solely by name; describe more complex techniques in the Methods section.*
☐	☒	A description of all covariates tested
☐	☒	A description of any assumptions or corrections, such as tests of normality and adjustment for multiple comparisons
☐	☒	A full description of the statistical parameters including central tendency (e.g. means) or other basic estimates (e.g. regression coefficient) AND variation (e.g. standard deviation) or associated estimates of uncertainty (e.g. confidence intervals)
☐	☒	For null hypothesis testing, the test statistic (e.g. *F*, *t*, *r*) with confidence intervals, effect sizes, degrees of freedom and *P* value noted<br>*Give P values as exact values whenever suitable.*
☐	☒	For Bayesian analysis, information on the choice of priors and Markov chain Monte Carlo settings
☒	☐	For hierarchical and complex designs, identification of the appropriate level for tests and full reporting of outcomes
☐	☒	Estimates of effect sizes (e.g. Cohen's *d*, Pearson's *r*), indicating how they were calculated

Our web collection on statistics for biologists contains articles on many of the points above.

Software and code

Policy information about availability of computer code

Data collection	DNA methylation data were generated using the Illumina Infinium MethylationEPIC BeadChip array. BeadArrays were scanned using Illumina iScan scanners. Data was processed in R using the minfi package.
Data analysis	The code to identify fCpG loci and to infer the stem cell dynamics of individual crypts was written in python (v3.9.2) and can be obtained on GitHub through https://github.com/CalumGabbutt/flipflop.git. Packages used include: Cython (v0.29.21), dynesty (v1.0.1), joblib (v1.0.0), numpy (v1.19.5), pandas (v1.1.5), scipy (v1.5.4). The GLM to infer differences between tissue types was written in python (v3.9.2) and used pystan (v2.19.1.1). The agent-based spatial modelling framework of the crypt is available at https://github.com/MathOnco/ticktockspatialmodel.git. The blood simulations illustrating how the methylation distribution changes following a rapid clonal expansion can be obtained, along with sample simulation results, at https://github.com/MathOnco/ticktockblood.git. A GUI compatible with most operating systems is accompanied to allow for rapid evaluation of different parameters.

For manuscripts utilizing custom algorithms or software that are central to the research but not yet described in published literature, software must be made available to editors and reviewers. We strongly encourage code deposition in a community repository (e.g. GitHub). See the Nature Research guidelines for submitting code & software for further information.

Data

Policy information about availability of data

All manuscripts must include a data availability statement. This statement should provide the following information, where applicable:

- Accession codes, unique identifiers, or web links for publicly available datasets
- A list of figures that have associated raw data
- A description of any restrictions on data availability

Raw and processed Illumina EPIC methylation array data collected in the process of this study are available at the European Genome-Phenome Archive (EGA) with

study accession number EGAS00001005514. Figures 2, 4 & 5 are associated with this data. The oscillatory CpG loci identified and the associated beta values of intestinal and endometrial samples are presented in supplementary tables 1 and 2 respectively.

Field-specific reporting

Please select the one below that is the best fit for your research. If you are not sure, read the appropriate sections before making your selection.

☒ Life sciences ☐ Behavioural & social sciences ☐ Ecological, evolutionary & environmental sciences

For a reference copy of the document with all sections, see nature.com/documents/nr-reporting-summary-flat.pdf

Life sciences study design

All studies must disclose on these points even when the disclosure is negative.

Sample size	The numbers of samples were chosen based on tissue availability to allow comparisons between tissues and within tissues. The sample size was sufficiently large to develop the methods in this paper.
Data exclusions	One colon sample was excluded from the analysis as the histogram of oscillatory CpG loci beta values lacked a clonal peak near 100% methylated, suggesting contamination of non-epithileal (non-clonal) cells.
Replication	No formal attempt was made to replicate the results of this study because of the inherent limited availability of appropriate human samples. However, we studied multiple samples from different individuals, and analyzed multiple independent GEO data sets with our algorithms.
Randomization	Randomization was not possible in the study because samples were collected in the process of standard clinical practice and sample classification was biology based and not subject to a random experimental assignment.
Blinding	Blinding was not attempted in the study because of the small sample sizes and investigators were aware of the biological classification of the samples. However, all samples were processed with standard commercial arrays and analyzed by the same algorithms.

Reporting for specific materials, systems and methods

We require information from authors about some types of materials, experimental systems and methods used in many studies. Here, indicate whether each material, system or method listed is relevant to your study. If you are not sure if a list item applies to your research, read the appropriate section before selecting a response.

Materials & experimental systems

n/a	Involved in the study	
☒	☐	Antibodies
☒	☐	Eukaryotic cell lines
☒	☐	Palaeontology and archaeology
☒	☐	Animals and other organisms
☐	☒	Human research participants
☒	☐	Clinical data
☒	☐	Dual use research of concern

Methods

n/a	Involved in the study	
☒	☐	ChIP-seq
☒	☐	Flow cytometry
☒	☐	MRI-based neuroimaging

Human research participants

Policy information about studies involving human research participants

Population characteristics	A full description of the age, sex, hereditary condition and whether a patient was diagnosed with cancer at the time of collection is provided in Supplementary Table 1.
Recruitment	Patients were not recruited for this study. The samples were excess tissues taken in the course of routine clinical care. The samples were chosen based on availability with a range of patient ages selected to explore this parameter.
Ethics oversight	Tissues were collected at the University of Southern California Keck School of Medicine from excess surgical samples taken in the course of routine clinical care, with Institutional Review Board approval (ref HS-18-00043).

Note that full information on the approval of the study protocol must also be provided in the manuscript.
